# Supplementary material for: Motivation as a mechanism underpinning exercise-based falls prevention programmes for older adults with cognitive impairment: a realist review
Source: BMJ Open. 2019 Jun 19;9(6):e024982. doi: 10.1136/bmjopen-2018-024982 (PMC6588958; doi:10.1136/bmjopen-2018-024982)
Supplement: Supplementary data [file bmjopen-2018-024982supp001.pdf]

## Supplementary Material

### Supplementary Table 1: RAMESES Realist Synthesis Checklist

| TITLE | | Reported in document Y/N/Unclear | Page(s) in document |
|---|---|---|---|
| 1 | In the title, identify the document as a realist synthesis or review | Y | 1 |
| ABSTRACT | | | |
| 2 | While acknowledging publication requirements and house style, abstracts should ideally contain brief details of: the study's background, review question or objectives; search strategy; methods of selection, appraisal, analysis and synthesis of sources; main results; and implications for practice. | Y | 2-3 |
| INTRODUCTION | | | |
| 3 Rationale for review | Explain why the review is needed and what it is likely to contribute to existing understanding of the topic area. | Y | 4-5 |
| 4 Objectives and focus of review | State the objective(s) of the review and/or the review question(s). Define and provide a rationale for the focus of the review. | Y | 5 |
| METHODS | | | |
| 5 Changes in the review process | Any changes made to the review process that was initially planned should be briefly described and justified. | N | |
| 6 Rationale for using realist synthesis | Explain why realist synthesis was considered the most appropriate method to use. | Y | 4-5 |
| 7 Scoping the literature | Describe and justify the initial process of exploratory scoping of the literature. | Y | 6 |
| 8 Searching processes | While considering specific requirements of the journal or other publication outlet, state and provide a rationale for how the iterative searching was done. Provide details on all the sources accessed for information in the review. Where searching in electronic databases has taken place, the details should include, for example, name of database, search terms, dates of coverage and date last searched. If individuals familiar with the relevant literature and/or topic area were contacted, indicate how they were identified and selected. | Y | 6 |
| 9 Selection and appraisal of documents | Explain how judgements were made about including and excluding data from documents, and justify these. | Y | 6-7 |
| 10 Data extraction | Describe and explain which data or information were extracted from the included documents and justify this selection. | Y | 7 |
| 11 Analysis and synthesis processes | Describe the analysis and synthesis processes in detail. This section should include information on the constructs analyzed and describe the analytic process. | Y | 7 |
| RESULTS | | | |
| 12 Document flow diagram | Provide details on the number of documents assessed for eligibility and included in the review with reasons for exclusion at each stage as well as an indication of | Y | 8 |

| | | | |
|---|---|---|---|
| | their source of origin (for example, from searching databases, reference lists and so on). You may consider using the example templates (which are likely to need modification to suit the data) that are provided. | | |
| 13 Document characteristics | Provide information on the characteristics of the documents included in the review. | Y | 8-9 |
| 14 Main findings | Present the key findings with a specific focus on theory building and testing. | Y | 10-16 |
| DISCUSSION | | | |
| 15 Summary of findings | Summarize the main findings, taking into account the review's objective(s), research question(s), focus and intended audience(s). | Y | 17 |
| 16 Strengths, limitations and future research directions | Discuss both the strengths of the review and its limitations. These should include (but need not be restricted to) (a) consideration of all the steps in the review process and (b) comment on the overall strength of evidence supporting the explanatory insights which emerged.<br>The limitations identified may point to areas where further work is needed. | Y | 17-19 |
| 17 Comparison with existing literature | Where applicable, compare and contrast the review's findings with the existing literature (for example, other reviews) on the same topic. | Y | 19-21 |
| 18 Conclusion and recommendations | List the main implications of the findings and place these in the context of other relevant literature. If appropriate, offer recommendations for policy and practice. | Y | 21 |
| 19 Funding | Provide details of funding source (if any) for the review, the role played by the funder (if any) and any conflicts of interests of the reviewers. | Y | 23-24 |

Supplementary Table 2: Example search strategy

| |
|---|
| Cochrane search strategy<br><br>In Title, Abstract, Keywords<br><br>(accidental fall OR fall* OR fall risk OR postural balance) AND (mild cognitive impairment OR dementia OR Alzheimer disease OR cognit* impair*) AND (Aged OR elderly OR elder OR frail OR old* OR seniors OR geriatric OR older adult*) AND (Exercise OR "physical exercise" OR "exercise therapy" OR "physical activity" OR balance OR "resist* training" OR strength) |

## Supplementary Table 3: Questions for selection and quality appraisal

| Selection Stage | Question |
|---|---|
| **Screening** | |
| Titles | Could this be about the strength and balance exercise component of falls rehabilitation in older adults with cognitive impairment in the community? |
| Abstracts | Could this material provide useful information about completing the strength and balance exercise component of falls rehabilitation in older adults with cognitive impairment in the community? |
| **Quality Appraisal of Articles** | |
| | Is the material cohesive? Does it tell a comprehensive story or is there a juxtaposition of ideas or isolated statements? |
| | What is the value of the evidence? Does it contribute to the topic area? |
| | What is the material's position in relation to the programme theory and general topic area? |

Supplementary Table 4: Quality appraisal of included studies

| Reference | Study Design | Is the material cohesive? | What is the value? | What is the position in relation to the programme theories? | Broad/ Narrow | Thick/ Thin |
|---|---|---|---|---|---|---|
| **Suttanon (2012)** | Qualitative (interview) | Yes [Hill (2009)] | Considerable value | Adds and supports to many MRTs | Broad | Thick |
| **Cedervall (2015)** | Qualitative (interview) | Yes [Cedervall (2010)] | Considerable value | Adds to many MRTs | Broad | Thick |
| **van Alphen (2016)** | Literature (meta-synthesis) | Yes | Valuable | Supports and refutes the same MRT | Broad | Thick |
| **Cedervall (2010)** | Qualitative (case study) | Yes [Cedervall (2015)] | Valuable | Supports and refutes the some MRTs | Broad | Thick |
| **Malthouse (2014)** | Qualitative (interview) | Yes | Valuable | Adds to many MRTs | Broad | Thick |
| **De Andrade (2013)** | Non-randomised trial | Yes | Valuable | Adds to a few MRTs | Narrow | Thick |
| **Hernandez (2010)** | Non-randomised trial | Yes | Valuable | Adds to a few MRTs | Narrow | Thick |
| **Hauer (2012)** | Randomised trial | Yes | Valuable | Adds to a few MRTs | Narrow | Thick |
| **Pitkala (2013)** | Randomised trial | Yes | Valuable | Adds to few MRTs | Narrow | Thick |
| **Garuffi (2013)** | Non-randomised trial | Yes | Valuable | Adds to many MRTs | Broad | Thin |
| **Blankevoort (2010)** | Literature (systematic) | Yes | Valuable | Neither adds nor detracts from MRTs | Broad | Thin |
| **Burton (2015)** | Literature (meta-analysis) | Yes | Valuable | Limited in adding to MRTs development | Broad | Thin |
| **Ries (2010)** | Non-randomised trial | Yes | Valuable | Adds to many MRTs | Broad | Thin |
| **Stubbs (2014)** | Literature (systematic) | Yes | Valuable | Adds to many MRTs | Broad | Thin |
| **Huger (2009)** | Protocol | No | Limited value | Adds to few MRTs | Broad | Thin |
| **Frederiksen (2012)** | Abstract | Yes | Limited value | Relevant to few MRTs but cannot support or refute | Broad | Thin |
| **Hauer (2006)** | Literature (systematic) | Yes | Valuable | Adds to few MRTs | Narrow | Thin |
| **Chan (2015)** | Literature (meta-analysis) | Yes | Valuable | Adds to few MRTs | Narrow | Thin |
| **Christofoletti (2007)** | Literature (systematic) | Yes | Limited value | Adds to few MRTs | Narrow | Thin |
| **Liu-Ambrose (2009)** | Literature (non-systematic) | Yes | Limited value | Adds to few MRTs | Narrow | Thin |
| **Shimada (2014)** | Abstract | Yes | Limited value | Adds to few MRTs | Narrow | Thin |
