# Supplementary material for: Motivation as a mechanism underpinning exercise-based falls prevention programmes for older adults with cognitive impairment: a realist review
Source: BMJ Open. 2019 Jun 19;9(6):e024982. doi: 10.1136/bmjopen-2018-024982 (PMC6588958; doi:10.1136/bmjopen-2018-024982)
Supplement: Author's manuscript [file bmjopen-2018-024982.draft_revisions.pdf]

BMJ Open

BMJ Open is committed to open peer review. As part of this commitment we make the peer review history of every article we publish publicly available.

When an article is published we post the peer reviewers' comments and the authors' responses online. We also post the versions of the paper that were used during peer review. These are the versions that the peer review comments apply to.

The versions of the paper that follow are the versions that were submitted during the peer review process. They are not the versions of record or the final published versions. They should not be cited or distributed as the published version of this manuscript.

BMJ Open is an open access journal and the full, final, typeset and author-corrected version of record of the manuscript is available on our site with no access controls, subscription charges or pay-per-view fees (http://bmjopen.bmj.com).

If you have any questions on BMJ Open's open peer review process please email info.bmjopen@bmj.com

BMJ Open

Motivation as a Mechanism Underpinning Exercise-based Falls Prevention Programmes for Older Adults with Cognitive Impairment: A Realist Review

Journal:	*BMJ Open*
Manuscript ID	bmjopen-2018-024982
Article Type:	Research
Date Submitted by the Author:	25-Jun-2018

Complete List of Authors:	Booth, Vicky; University of Nottingham School of Medicine,<br>Harwood, Rowan; Health Care of Older People, Nottingham University Hopsitals NHS Trust<br>Hancox, Jennie; University of Nottingham School of Medicine, Division of Rehabilitation and Ageing<br>Hood-Moore, Victoria; University of Nottingham School of Medicine, Division of Rehabilitation and Ageing<br>Masud, Tahir ; Nottingham University Hospital, Geriatric<br>Logan, Phillipa; The University of Nottingham, School of Medicine, Division of Rehabilitation and Ageing

Keywords:	Dementia < NEUROLOGY, REHABILITATION MEDICINE, SPORTS MEDICINE

SCHOLARONE™
Manuscripts

Motivation as a Mechanism Underpinning Exercise-based Falls Prevention Programmes for Older Adults with Cognitive Impairment: A Realist Review

Authors

Vicky Booth, Rowan H Harwood, Jennie E Hancox, Victoria Hood-Moore, Tahir Masud, and Pip A Logan.

Authors Address

Vicky Booth, School of Medicine and Health Sciences, University of Nottingham, Victoria.Booth@nottingham.ac.uk (corresponding author)

Rowan H Harwood, School of Medicine and Health Sciences, University of Nottingham, ntzrhh@nottingham.ac.uk

Jennie E Hancox, University of Nottingham, mszjeh@nottingham.ac.uk

Victoria Hood-Moore, School of Medicine and Health Sciences, University of Nottingham, mczvlh@nottingham.ac.uk

Tahir Masud, General Medicine, Nottingham University Hospitals NHS Trust, Tahir.Masud@nuh.nhs.uk

Pip A Logan, School of Medicine and Health Sciences, University of Nottingham, Pip.Logan@nottingham.ac.uk

For peer review only - http://bmjopen.bmj.com/site/about/guidelines.xhtml

Abstract

Objectives

The aim of this review is to identify and evidence the mechanisms underlying falls prevention interventions, in older adults with cognitive impairment, to aid intervention development and implementation. The research question is how does an exercise intervention work for people with cognitive impairment, in what circumstances, and why.

Design

A realist review of the literature.

Setting and Participants

Publications reporting exercise-based interventions for adults with dementia (any level of cognitive impairment) in the community were included.

Interventions

Exercise-based interventions.

Outcome measures

A "rough programme theory" (a preliminary model of how an intervention works) was tested against published literature identified from a comprehensive search strategy. Data was collected according to elements of the programme theory and not isolated to outcomes.

Results

Motivation is a core element of the programme theory describing participation in exercise-based interventions for people with cognitive impairment. When the individual thinks that exercise will be beneficial they will access supportive mechanisms to enable it. Supportive mechanisms include having a "gate-keeper", such as a therapist or carer, who shares or takes responsibility for the perception of exercise as beneficial, enabling the person with dementia to access and participate in exercise programmes. A lack of access to support had a detrimental effect on adherence and

For peer review only

For peer review only - http://bmjopen.bmj.com/site/about/guidelines.xhtml

participation in exercise. Motivational mechanisms were particularly relevant for older adults with mild to moderate dementia, where the exercise intervention was multicomponent, in a preferred setting, at the correct intensity and level of progression, correctly supported and considered, and flexibly delivered.

Conclusion

This is the first realist literature review in falls prevention research. Many aspects of the motivational mechanisms identified in this review have parallels with self-determination theory. Clinically relevant recommendations were derived and will be used to further develop and test a motivationally considered exercise-based falls intervention for people with mild dementia.

Registration

PROSPERO CRD42015030169.

Keywords

Dementia; Cognitive Impairments; Exercise; Accidental Falls.

Word count

3239

Strengths and Limitations of this study

- Traditional systematic reviews report a dearth of evidence for falls interventions for this population indicating the need for novel approaches to evidence synthesis.
- This is the first realist literature review in falls prevention research.
- Approaching the evidence in this manner provides a valuable addition to the research on the exercise components of falls intervention programmes for older adults with mild cognitive impairment.

For peer review only - http://bmjopen.bmj.com/site/about/guidelines.xhtml

- A phased, iterative and interactive search process was identified and adapted during the review process that included all studies involving people with dementia, participating in an exercise-based intervention in a community setting.

Introduction

Falls prevention is a complex intervention. In the general older adult population there is conclusive and robust evidence that falls risk can be reduced [1] however the same certainty cannot be provided to people with cognitive impairment [2]. People with cognitive impairment, such as dementia, have a high risk of falling [3-5], which frequently results in injury or hospital admission. However, clinical guidelines are unable to recommend evidence-based interventions due to the paucity of research in this field [6].

Exercise has been shown to reduce falls risk [1]. Anecdotal evidence suggests that older adults with dementia are poor adherers to exercise programmes due to problems with memory, executive function and co-morbidities. Research is needed to explore the contextual factors and mechanisms associated with exercise engagement in older adults with dementia.

Realist synthesis is increasingly used for evaluating evidence for complex health and social interventions [7]. A realist review explores how underlying mechanisms (M) might be 'triggered' in the context (C) of a particular therapy in a particular population to produce an intended or unintended outcome (O). Mechanisms are further subdivided between resources, and responses [8]. Theory is generated and described through this Context-Mechanism-Outcome (CMO) heuristic [9]. CMO Configurations (CMOCs) can be interlinked, creating chains of possibilities and generating theories to explain why a particular outcome occurs with a specific intervention [10]. Interlinking CMOCs can be clustered together to form middle-range theories (MRT), and in turn, the programme theory or model of how an intervention works [7]. Realist methods encourage the

For peer review only - http://bmjopen.bmj.com/site/about/guidelines.xhtml

incorporation of data from a range of sources, accommodating complexity that is inherent in health research [11].

Traditional systematic reviews examine the effectiveness of a defined intervention ('does it work?'), as opposed to exploring the underlying mechanisms, which, in theory, may be more generalizable when studying complex interventions in heterogeneous populations. A detailed rationale for completing a realist review in this field has been published and reported in the lead authors PhD thesis [12, 13]. There are a limited number of studies in this field, which have used different research methods [14]. Dementia populations vary according to level of impairment (mild to severe) and diagnosis (e.g., Alzheimer's disease, frontotemporal dementia). Therefore, what is relevant to one individual might be different for another. Developing a theoretical framework to rationalise and explain the key principles behind an intervention will aid its development and implementation [15] and take into account the complexity of the population.

The objective of this review was (i) to identify the underlying programme theory for motivation to undertake exercise-based interventions targeted at individuals who have been identified as at risk of falling and who have cognitive impairment, and (ii) to explore how and why that intervention reduces falls in that population. The aim was to produce a list of recommendations that could be used clinically or for further intervention development.

Methods

Study Design

The review followed the stages identified by Pawson et al [16] including; i) articulating key rough programme theories to be explored, ii) searching for relevant evidence, iii) appraising the quality of

For peer review only - http://bmjopen.bmj.com/site/about/guidelines.xhtml

evidence, iv) extracting the data, and v) synthesising evidence. A detailed protocol has been published [12] and guidance for publication of realist synthesis has been followed [17] and reported.

Scoping

Initial scoping was based on prior knowledge of the falls literature [14] and extensive clinical experience. The purpose was to clarify the aims of the review, develop an initial rough programme theory, and direct the search strategy for the main review.

Search processes

A phased literature search was conducted. An electronic search was completed of databases: EMBASE, MEDLINE, CINAHL, the Cochrane Library, PsycINFO, and PEDRO. Keywords and MESH headings were adapted according to the database used and included: *accidental falls, falls rehabilitation, falls prevention, exercise, dementia, cognitive impairment*.

The "*iterative and interactive"* [18] search process evolved during the review, utilising "cited-by" and manual reference list search to find additional primary evidence that was required to refine a particular aspect of the programme theory. The "cited-by" search was completed using Google Scholar for studies that had cited one of the qualitative papers [19]. The reference list of a systematic review [18] was manually searched. Material was chosen for; i) focus on dementia population, ii) qualitative methods, and iii) reporting experiences of completing an exercise or physical-activity intervention. The search results were screened by the researcher (VB) who documented the number of articles retrieved during each search stage using EndNote reference management software.

Selection and appraisal of documents

As is acceptable in realist reviews, material was included regardless of study method but had to focus on an exercise intervention, be published in English and involve community-based participants

For peer review only - http://bmjopen.bmj.com/site/about/guidelines.xhtml

or interventions (such as those based in a hospital). Reasons for exclusion were documented and a second researcher (PL or RH) consulted to aid decision making when required.

Titles and abstracts were screened according to relevance of the material to the synthesis aims [20] (Supplementary Table 1). Relevance and rigour were assessed to define the quality of the included material using a series of judgements [18].

The full-text for eligible studies was assessed for quality and extraction of data. A random sample of 10% of the materials was selected and assessed by the stakeholder group comprising rehabilitation and medical clinicians and academics.

Data extraction

Data were extracted based on relevance to the aims of the review and the rough programme theory. Data were sought that substantiated, refined or refuted the theories. Data regarding contextual characteristics were documented. Relevant material was highlighted, labelled, and recorded [21]. NVivo software and Excel was used to record and code the extracted data.

Analysis and synthesis process

Extracted material was coded as context, mechanism or outcome, and judgements regarding how this influenced the CMOCs recorded through annotations. Codes were initially allocated to each MRT within the rough programme theory, and as each article was processed, these codes were iteratively adapted according to the new material. Material that was relevant to more than one MRT were coded accordingly with links across theories.

Three waves of searching, analysis and synthesis occurred to direct the next stage of the review. Emerging findings were documented and then discussed with a stakeholder group.

For peer review only - http://bmjopen.bmj.com/site/about/guidelines.xhtml

Results

Document flow diagram

The initial search identified 1954 papers (Figure 1). Sixty-one papers were screened for inclusion. Sixteen papers from the initial search were not included as theoretical saturation had been reached (e.g., no new findings were emerging with the consideration of new papers). The iterative search identified a further four papers.

Figure 1: PRISMA Flow diagram for review documents.

Document characteristics

Twenty-one papers contributed data to the motivational mechanisms [19, 22-41]. The papers varied in methodological design including; qualitative studies (n=4) [19, 25, 26, 36], literature reviews (n=8) [23, 24, 27, 28, 31, 35, 40, 41], randomised (n=2) [32, 37] and non-randomised trials (n=4) [22, 30, 33, 38], protocols (n=1) [34], and conference abstracts (n=2) [29, 39].

Contextual information, including the levels of cognitive impairment and the type, dose, and setting of the interventions, are summarised (Table 1).

Quality Appraisal

All of the papers were quality appraised and critiqued according to cohesion, value, position against other material and the rough programme theory (Supplementary Table 2).

For peer review only - http://bmjopen.bmj.com/site/about/guidelines.xhtml

Table 1: Characteristics of included studies with emphasis on the intervention [13].

Reference	Cognitive level	Type of intervention	Dose (*total hours*)	Setting
**Pitkala (2013)**	AD (67% mod/severe).	Intense, long-lasting physical exercise by dementia specialist physiotherapists in either home (HE) or group (GE) vs control (CG).	HE = 1 hr, 2 p/w, 12 mth (*104*); GE = 4 hr, 2 p/w, 12 mth (*416*).	Community, Finland.
**Shimada (2014)**	Amnesic MCI.	Multi-component group exercise by physiotherapists.	90 min, 2 p/w, (n=40 sessions) 10 mth (*130*).	Community, Japan.
**Hernandez (2010)**	AD (mild/mod).	Supervised group programme of regular physical activity.	1 hr, 3 p/w, 6 mth (*78*).	Community, Brazil.
**Hauer (2012)**	Dementia (mild/mod) (MMSE 21.7).	Progressive resistance and functional group training programme supervised by a qualified instructor.	2 hr, 2 p/w, 3 mth (*52*).	Community, Germany.
**de Andrade (2013)**	AD (mild/mod).	Aerobic, strength, flexibility, balance and cognitive exercises.	1 hr, 3 p/w, 16 wk (*48*).	Community, Brazil.
**Garuffi (2013)**	AD.	Resistance training.	1 hr, 3 p/w, 16 wk (*48*).	Community, Brazil.
**Huger (2009)**	Dementia (mild/mod, MMSE 17–26).	Progressive resistance and functional training including attention-depending motor-cognitive demands in small groups.	2 hr, 2 p/w, 12 wk (*48*).	Community, Germany
**Frederiksen (2012)**	AD (mild/mod).	Aerobic exercises (exercise machines) by physiotherapist.	1 hr, 3 p/w, 14 wk (*42*).	Community, Denmark.
**Ries (2010)**	AD (mean MMSE 23.2).	Balance exercise group with 2:1 supervision.	45 min, 2 p/w, 8 wk (*12*).	Community, USA.
**Suttanon (2012)**	AD (mild/mod).	Tailored home-based balance exercise by physiotherapist.	6 visits, 6 mth (*6*).	Community, Australia.
**Cedervall (2015)**	AD (mild).	Physical activity.	Range = "never" to 1 hr p/d.	Community, Sweden.
**Cedervall (2010)**	AD (mild).	Outdoor walking.	"Routine".	Community, Sweden.
**Malthouse (2014)**	AD (MMSE 18 to 21).	Physical activity.	"Routine".	Community, UK.
**Hauer (2006)**	Dementia (mod/severe)	Physical training.	Range=30–150 min, 2–7 p/w, 2–30 wk (*2-525*).	Mixed.
**Burton (2015)**	Dementia (MMSE 18.9).	Exercise or physical activity programme.	Range = 1-5 p/w, 3-12 mth (*13-260*).	Mixed.
**Blankevoort (2010)**	Dementia.	Physical activity.	Various.	Mixed.
**Chan (2015)**	Cognitive impairment.	Physical exercise.	Various.	Mixed.
**Stubbs (2014)**	Dementia.	Physical activity.	Various.	Community, mixed.
**Liu-Ambrose (2009)**	n/a.	Aerobic and resistance training.	2 p/w.	n/a
**van Alphen (2016)**	AD.	Physical activity.	n/a	Community, mixed.
**Christofoletti (2007)**	Dementia (mixed).	Motor intervention.	Various.	Mixed.

For peer review only - http://bmjopen.bmj.com/site/about/guidelines.xhtml

Motivation Programme Theory

The programme theory elaborated from the literature comprise eleven MRTs which explain how exercise is successfully undertaken. Motivation emerged as a well populated mechanism [19, 22-41] and is described in detail. Two key mechanisms were shown to be operating; a) perceived benefit and b) support.

"Perception of benefit" response-mechanism

An older adult (C^{1}), with dementia (C^{2}), with the belief that exercise is advantageous (C^{3}) or a positive attitude to exercise (C^{4}), will perceive the benefit ($M^{response1}$) of completing (O^{1}) an exercise intervention ($M^{resource}$).

An older adult (C^{1}), with dementia (C^{2}), with either a carer or themselves having previous experience of being active and participating in exercise (C^{5}), and with a perception that exercise is beneficial (C^{6}), will feel encouraged ($M^{response2}$) to complete (O^{1}) an exercise intervention ($M^{resource}$).

An older adult (C^{1}), with dementia (C^{2}), who has the ability to comprehend (C^{7}) and identifies physical or functional changes (C^{8}) will perceive the benefit ($M^{response1}$) from completing (O^{1}) an exercise intervention ($M^{resource}$).

An older adult (C^{1}), with dementia (C^{2}), will perceive benefit ($M^{response1}$) from completing (O^{1}) an exercise intervention ($M^{resource}$) if they have an external supporter who either: understands the benefits of exercise; can prompt regarding the benefits of exercise; enables the person with dementia to compare themselves with others (C^{9}).

An older adult (C^{1}) with dementia (C^{2}) who has a carer who perceives the benefit the person with dementia doing exercise out-weighs the risk of doing exercise or the burden of extra care duties (C^{10}) will provide support ($M^{response3}$) for the participation (O^{1}) in an exercise programme ($M^{resource1}$) or routine physical activity ($M^{resource2}$).

An older adult (C^{1}) with dementia (C^{2}) who has a carer who perceives benefit on mood (C^{11}), behaviour (C^{12}), body weight (C^{13}), flexibility (C^{14}), ageing (C^{15}) and enjoyment of everyday life (C^{16}) for that older adult doing exercise (C^{17}) will provide support ($M^{response3}$) for the participation (O^{1}) in an exercise programme ($M^{resource1}$) or routine physical activity ($M^{resource2}$).

Box 1: Perception of benefit CMOCs [13]

For peer review only - http://bmjopen.bmj.com/site/about/guidelines.xhtml

The perception or feeling of benefit emerged from seven of the included papers [19, 23, 25, 26, 34, 36, 41]. Perceiving the benefit of an exercise-based intervention could be either a response-mechanism or context, moving within the CMOC depending upon the individual and other context components [19]. Interpretation of Suttanon et al [19] indicates the perception of benefit could be a response-mechanism that is operating when the person with cognitive impairment has "*prior experience of being active, participating in exercises, and perceiving benefits of general exercise*" (p1180, [19]). These context features are relevant for both the participant and carer [19, 25]. Understanding an individual's previous experience of exercise and their perceptions of it can allow tailoring of approaches [19]. That perception or belief of the benefits of exercise may also be a characteristic or feature (context) of the person with cognitive impairment or their carer, which therefore encourages them to participate in the exercise intervention [17].

Recognition of improvements or changes in physiological-responses (for example in function or physical ability) reinforce an individual's perception of benefit [23]. Identification of benefit is important for both participation and the maintenance of an intervention [25, 26]. Huger et al [34] identified that persons with cognitive impairment can experience "*multiple problems*" which could include lack of comprehension. This would influence ability to identify benefits from completing exercise, and whilst this statement seems particularly negative, it could be interpreted as a context component for certain individuals, rather than a general characteristic of all older adults with cognitive impairment.

Synergy is required between carers' understanding and support, ability to address barriers to exercise, and the participants' comprehension [25, 41]. Perceiving the health benefits for others also generates the support and encouragement that prompts participation (e.g., an individual walking his dog [25]). The feeling of encouragement could also come from being able to compare

For peer review only - http://bmjopen.bmj.com/site/about/guidelines.xhtml

themselves with others that have the same diagnosis, but only in the context where the older adult with dementia was doing well [26].

The carer's perception and belief in the benefit of exercise must out-weigh the risk, care burden or adaption required to complete the exercise [25, 36]. Negative connotations associated with exercising (e.g., reminder of inability to do previously enjoyable activities or potential deterioration), or adaptions or changes to routines or daily lives that are required to support the physical activity, are destructive to the perception of benefit for both the person providing the support and the person with dementia [25]. Concern can be both facilitator and barrier to engagement in exercise, requiring a judgement between not staying mobile and healthy, against concern about getting lost or falling [36].

There was a range of benefits perceived from completing exercise which were not limited to health outcomes. A contentious benefit was an attempt to re-establish previous activities or the "person" that came before the dementia diagnosis or progression of dementia [25]. Cedervall and Aberg [25] reported this perception as coming from the person providing the support. The consideration of how exercise might influence dementia or benefit falls risk was not directly reported [36].

Support

An older adult (C^{1}) with dementia (C^{2}) who is supervised by trained personnel ($M^{resource1}$) who give clear/repeated instructions (C^{3}), optimally progress the exercises (C^{4}), provide the amount of supervision required by that individual and their needs (C^{5}), and understands the needs of persons with dementia (C^{6}) will feel supported ($M^{response1}$) to complete (O^{1}) an exercise programme ($M^{resource2}$).

An older adult (C^{1}) with dementia (C^{2}) who is being supported ($M^{response1}$) by a professional person ($M^{resource1}$) who can time-manage (C^{7}), is knowledgeable (C^{8}), firm but encouraging (C^{9}), is kind/friendly/supportive (C^{10}), who understands dementia (C^{11}) and can develop a rapport with the individual (C^{12}), will do an exercise programme (O^{1}).

An older adult (C^{1}) with dementia (C^{2}), who has a carer (C^{13}) who perceives a benefit from the older adult doing exercise (C^{14}) and can provide transport (C^{15}), a positive attitude (C^{16}), practical considerations (C^{17}),

For peer review only - http://bmjopen.bmj.com/site/about/guidelines.xhtml

For peer review only

supportive strategies (C^{18}), and/or assistance (C^{19}) will feel supported ($M^{response1}$) to complete (O^{1}) an exercise programme ($M^{resource1}$) or routine physical activity ($M^{resource2}$).

An older adult (C^{1}) with more severe dementia (C^{20}) will require more support ($M^{response1}$) to successfully participate (O) in exercise programme ($M^{resource1}$) or routine physical activity ($M^{resource2}$).

An older adult (C^{1}) with dementia/AD (C^{2}) who has a carer (C^{13}) who receives information (C^{21}) and on-going support (C^{22}) from the therapist/staff ($M^{resource1}$) to enable them to support ($M^{response1}$) the participation/completion of an exercise programme of the person with dementia (O^{1}).

An older adult (C^{1}) with dementia (C^{2}) who wants to exercise in a group (C^{23}), will feel supported ($M^{response1}$) to complete (O^{1}) a group exercise programme ($M^{resouce1}$) or group physical activity ($M^{resource2}$).

An older adult (C^{1}) with dementia (C^{2}) who has a poorer ability to understand and learn new information (C^{24}), who has not exercised previously (C^{25}), has ill-health (C^{26}), or has regular holidays (C^{27}), will not access the required support needed to exercise ($M^{resource1}$) and therefore will not feel supported ($M^{response1}$) to complete an exercise programme(O^{1}).

Box 2: Support CMOCs [13]

Support was a mechanism of motivation reported by fourteen papers [19, 23-29, 34, 36-38, 40, 41]. The support could be provided through supervision [27, 34], practical measures [25], strategies such as making or maintaining routines [26] or through emotional support [26, 34]. There were many references to who provided the support and how it was given.

Supervision was discussed as a component of support [27]. Supervision by trained personnel "*met the special needs of persons with cognitive impairment*" (p153, [27]) by; giving clear and repeated instructions, optimally progressing the programme, and providing the amount of supervision required depending upon their ability to understand and learn new information [27]. Training instructors or supervisors provide more than just formal support during an intervention [34] and were influential in the commencement, participation and maintenance of exercise [19]. Key characteristics of the professional person were identified [19]. An ability to "*understand my*

For peer review only - http://bmjopen.bmj.com/site/about/guidelines.xhtml

For peer review only

problem" (p172, [36]) also emerged as important, particularly in regards to dementia. This facilitated rapport development between supporter and person with dementia, which included a relationship built on personal information [38] and trust [41].

Carers were a frequently discussed and important component with the support they provided [40]. The role of the carer was described by Malthouse and Fox [36] as "*facilitators to activity*" and "*gate-keepers*". There were many ways in which the carers provided support including the avoidance of stressful or negative situations [36], providing transport [22, 33], promoting a positive attitude [29], organising the practical arrangements [25], employing specific strategies [26], providing additional assistance [40] and counteracting the "*loss of initiation and motivation*" [41] that were specific to that older adult with dementia and their situation. The promotion of exercise either in the practical or emotional sense, implies an underlying assumption or perception that the activity is beneficial for the individual or themselves as a carer. However, this is a complex interaction illustrated by contradictory data indicating that carers did [28, 37] or did not [36, 37, 40, 41] receive encouragement, benefit or reduced distress from providing support for the person with cognitive impairment.

Carers provided varying levels of support that were tailored to the individual [19, 25]. Carer involvement was integral to programme delivery in one study [19]. The carer and their support was more influential the more severe the cognitive impairment and may account for why persons with severe dementia were still able to engage in interventions [40]. However, it was highlighted how complex the support component is, particularly as impairments progress [40]. Van Alphen et al [41] suggest that because persons with dementia require care and support that they are more influenced by support as a variable within an intervention. The carers themselves also require support, with information identified as a resource-mechanism to enable the support to take place. Carers require information from the intervention staff to enable their support of the person with dementia [19, 41].

For peer review only - http://bmjopen.bmj.com/site/about/guidelines.xhtml

A group setting for the intervention provided support from both the trained staff and social aspects [30, 33]. Individuals in the group contribute to the intervention, providing understanding of the issues and experiences of someone with dementia [36, 41]. Positive results from the social aspect of a group intervention were reported by some studies directly [19] and indirectly [31]. However, this was not consistent across all material [30]. Differences in participation and outcomes could be explained by the personality and preferences of the individuals. Some individuals will have had a strong opinion on attending groups of people with dementia and this in itself will have influenced their participation [19, 32, 37].

Lack of support resulted in poor adherence or participation in exercise [24]. Contexts that contributed to poor participation included lack of previous exercise experience, ill-health and holidays [24]. Lack of support is also attributed to poor effectiveness in trials of certain interventions [31] and as a barrier to physical activity [41].

Intervention Contextual Characteristics

An older adult (C[1]) with mild to moderate dementia (C[2])

Interventions can be in either a home or group setting according to the preferences/wishes of the individual and their carer/spouse (C[3]) (a home setting might be preferable for those wanting individual support from the intervention staff, or a group setting might be preferable for those wanting carer respite, or opportunities for social contact or engagement).

Interventions that are multicomponent combining physical (including strength/resistance, balance, endurance/mobility, aerobic) and cognitive exercises (C[4]), at the correct intensity and level of progression (C[5]), supported in the correct way by suitable staff and materials (interaction, communication and connection) (C[6]), and with consideration for speed of initiation, length of intervention, encouragement of active lifestyle and enjoyment (C[7]).

Intervention that is provided flexibly (C[8]), for 6-12 months (C[9]), 2-3 times a week (C[10]), for minimum 15-20 minutes or whatever can be done or fit in with routine (C[11]).

Box 3: Intervention Contextual CMOCs [13]

For peer review only - http://bmjopen.bmj.com/site/about/guidelines.xhtml

Many studies included participants with a range of cognitive impairment levels, from mild to moderate, with only two featuring moderate or severe dementia [31, 37]. Generalising across cognitive levels is not appropriate as improvements found from an intervention at one stage may not be found in another [31, 32]. A "*critical period*" for improvement within the cognitive impairment continuum was suggested [42].

Reports conflicted regarding which setting was preferable, home [19] or group [37]. Characteristics of the different settings suited the preferences and wishes of different individuals and their situations. A solution where multiple settings (home and group) and locations (inside and outside) for the same intervention was suggested [19, 41].

A range of interventions were included. Intensity and progression were important [22, 24, 38]. The influence of the intervention staff (interaction, communication and connection) [19, 36, 38] and the materials provided [19] were considered a component and asset of the intervention. Particular recommendations to consider the speed of initiation and length of intervention were identified [36]. Certain types of activity were more enjoyable for different individuals, as was the inclusion of certain types of exercise into the routine of daily life [26, 36, 37, 41].

The "dose" of an intervention is a combination of frequency, duration, and intensity. A range of doses was reported (Table 1). Overall, the optimal dose for an exercise intervention for persons with cognitive impairment has not been defined [30], is poorly understood [42], but is important [28]. The concept of "routine", both in content (such as a daily walk) and duration (such as fitting into daily life [19]) was highlighted [25, 26, 36, 41]. A flexible approach limited absences, particularly in consideration of the mood [36] or other health conditions [19] of the participant or their carer.

For peer review only - http://bmjopen.bmj.com/site/about/guidelines.xhtml

Discussion

Summary of findings

Motivation is a core element of the programme theory describing participation in exercise-based interventions for people with cognitive impairment. When an older person with mild to moderate cognitive impairment has the perception that exercise will be beneficial they can utilise supportive mechanisms and contexts to complete an exercise programme. Support as a motivational mechanism requires a "*gate-keeper*", such as a therapist or carer, who shares or takes responsibility for the perception of exercise as beneficial, thereby enabling the person with dementia to access and participate in exercise programmes. Having a perception of benefit is both a mechanism and contextual feature within this programme theory. A lack of access to support had a detrimental effect on adherence and participation in exercise.

Strengths and limitations

This review progress the falls prevention research by using a novel method and approach. The main strength of this review is the successful completion of realist rationale in a historically positivist research field which demands causal probabilities but which contains practice and policy directed by evidence. The realist review methodology was well-suited to the research question. Consideration of the mechanisms underpinning exercise-based interventions allowed development and extrapolation of the theoretical rationale. Exploring and documenting context components allows individualisation.

Transparency is encouraged in realist methods of enquiry and is evident in this review regarding the interpretation of material being influenced by the researcher. The researcher is acknowledged in this review and, whilst considered from a traditional systematic review method as producing the potential for bias, has assisted in the theory development and interpretations. Detailed recognition of underlying or "*hidden*" mechanisms and understanding of the CMOCs was strengthened by the

For peer review only - http://bmjopen.bmj.com/site/about/guidelines.xhtml

experience of the main researcher (as a physiotherapist) and the stakeholder group from their work with older people and falls prevention.

A micro (interpersonal) level [43] was the focus for the MRT's and overall programme theory, with the review not expanding to consider meso (institutional) or macro (government and policy) levels of social structure [43]. The review also did not base the initial rough programme theory on any overarching motivational theories (such as Self-Determination Theory [44, 45]). Theoretical frameworks are typically consulted to structure realist reviews (e.g., search strategies and data analysis) [43, 46]. This was not done in this review and is potentially a limitation. However, the programme theory and its subsequent recommendations are very specific and should be viewed as a considerable strength.

The quality and content of the evidence available limited the review. Quantitative methods are more prevalent in research involving falls interventions with publications following specific reporting standards that does not encourage theoretical speculation. Information regarding participants and their influencing characteristics were rarely discussed. Greater contextual and resource information may be a product of recommendations for increased detail in reporting interventions (e.g., the TIDieR guidelines [47]).

Material relevant to the review may not have been identified. Further iterative searches and snow-ball searching was not completed in view of the restricted time and resources.

Future research directions

All of the materials included within the review described participants who had either completed regular physical-activity or the exercise-based intervention under study. The perspective of those not completing an exercise-based intervention must be considering for further programme theory refinement particularly considering the motivational mechanisms.

For peer review only - http://bmjopen.bmj.com/site/about/guidelines.xhtml

The review process has clearly directed the need for a realist evaluation to test the refined programme theory. A realist evaluation could utilise data from an existing exercise-based intervention in people with mild dementia and cognitive impairment to assist participation and adherence [48, 49].

Comparison with existing literature

Realist reviews have previously been undertaken to explore issues involving people living with dementia [50-54] and aid explanation in other health systems and complex interventions [55-59].

These findings relate to a wider literature and existing theories of motivation. Many of the CMOs identified in this review have parallels with self-determination theory (SDT; [60, 61]). SDT is a theory of motivation which focuses on the mechanisms by which the social environment created by significant others (e.g., therapists or carers) influences individuals' motivation to engage in specific behaviours (e.g., exercise). Previous SDT research (e.g., Murray et al., [62]) has focused on the communication style utilised by healthcare professionals (i.e., what they say and how they say it) and the extent to which this satisfies participants' basic psychological needs (for competence, autonomy and relatedness [61]). This review has highlighted communication strategies (i.e., developing a rapport with the individual, being firm but encouraging, and promoting optimal progression) similar to those considered as need-supportive, and associated with participant completion of exercise programmes. Thus, future intervention research within this population may want to consider drawing from SDT and training therapists to adopt a need-supportive communication style.

The 'perception of benefit' response-mechanism has parallels with the SDT mechanism of 'identified regulation'. Another key assumption of SDT is that there are qualitatively different reasons underlying behavioural engagement [63]. One of the more autonomous reasons for engaging in behaviour is identified regulation, which is when individuals engage in an activity because they

For peer review only - http://bmjopen.bmj.com/site/about/guidelines.xhtml

identify with the benefits. Similarly to the CMOs presented within the 'perception of benefit' section of the results, research based on SDT has found that identifying with the benefits is an important mechanism mediating the relationship between the social context created by significant others (e.g., therapist or carers) and individuals' engagement in exercise behaviour [64]. Previous research has suggested that SDT is a suitable framework for investigating exercise engagement of older adults [65], however, SDT has not been applied in research exploring exercise participation in individuals with cognitive impairment or dementia. This review extends current knowledge by highlighting a potential limitation with the applicability of the SDT construct of identified regulation to all individuals with dementia as some may not have the psychological capability or capacity to comprehend (C^7) the benefits of taking part in the exercise programme.

Achievement Goal Theory (AGT) [66, 67] is another theory of motivation which has similarities to the findings of this review, and is also is conceptually related to SDT. Results revealed comparison to others in a group setting as a motivating factor for older adults completing an exercise intervention but only when their performance is superior. AGT proposes that an important prerequisite for motivated behaviour is a desire to feel competent [68] and that individuals can be more or less task- or ego-involved. Individuals who operate a more task-involved goal perspective perceive themselves as successful when they try their best and improve their own performance [69]. In contrast, more ego-involved individuals compare their performance to others and feel successful only when their performance is superior [67]. Previous research suggests that although encouraging other-referenced comparisons may be a positive motivator in the short-term, in the long-term it can be associated with maladaptive outcomes such as lower levels of exercise participation [70]. Therefore, the inclusion of comparison against others in exercise interventions should be used with caution.

Making or maintaining routines was identified as a support mechanism associated with exercise programme completion. Similarly, a meta-analysis [71] found the creation of physical activity habits to offer a means to support maintenance of physical activity behaviours overtime. Thus, future

For peer review only - http://bmjopen.bmj.com/site/about/guidelines.xhtml

research looking to support the development of routine exercise in older adults with dementia may want to consider psychological theory on non-conscious processes, such as habit-formation in order to support long-term exercise completion.

Conclusion and recommendations

Older adults with mild to moderate cognitive impairment experience falls. Interventions, such as exercise, should be considered a resource that can positively influence this “outcome”, when used in the right circumstances or contexts. This realist review highlighted that consideration of the circumstances and underlying mechanisms for exercise-based interventions are important and could lead to greater success for future research, the individuals involved and their support networks. Recommendations for what types of exercise-based interventions for people with dementia, under what circumstances would aid motivation are provided (Table 2).

For peer review only

For peer review only - http://bmjopen.bmj.com/site/about/guidelines.xhtml

Table 2: Clinically relevant recommendations from the review results [13]

Focus	Recommendation
**Who**	Older adults with mild to moderate cognitive impairment
	If a person with dementia has the belief that exercise is advantageous, a positive attitude to exercise, the ability to understand the benefits of exercise or is able to identify the physical or functional changes from doing exercise, then they will perceive the benefit of doing exercise
	If a person with dementia perceives the benefit, they will participate in exercise-based intervention
**What**	Multicomponent exercise-based intervention that;<br>• combines physical (including strength/resistance, balance, endurance/mobility, aerobic) and cognitive exercises<br>• appropriately intensive and progressive<br>• supported by suitable staff (who can interact, communicate and connect) and materials<br>• considers speed of initiation, length of intervention, encouragement of active lifestyle and enjoyment<br>• is delivered in a flexible manner for at least 15-20 minutes (or whatever can become or fit in with routine) 2-3 times a week for 6-12 months<br>• can be delivered at home (for those wanting or needing 1:1 support from the intervention staff) or in a group (for those wanting carer respite, increase in habitual physical activity or socialising aspects)
**Circumstances**	Support can provide encouragement for completing an exercise-based intervention
	Sources of support can include but are not exclusively supplied by; trained intervention staff, carer, spouse, family member
	If support is being provided by trained intervention staff, then they should have professional competence including;<br>• time-management<br>• knowledgeable<br>• firm but encouraging<br>• kind, friendly and supportive<br>• understanding of the issues experienced by persons with dementia<br>• rapport development
	Trained intervention staff supporting an intervention should;<br>• provide clear and repeated instructions<br>• optimally progress the exercises<br>• provide the amount of supervision required by that individual and their needs<br>• understand the needs of persons with dementia
	If support is being provided by a carer, then the intervention should provide information and on-going support to enable them to continue
	Carers supporting an intervention should;<br>• perceive and understand the benefit of the person with dementia doing exercise<br>• provide transport or consider practical arrangements for access to the intervention<br>• have a positive attitude<br>• implement supportive strategies and/or assistance in the manner required by the person with dementia
	If the carers or supporters perception of the benefits of doing exercise out-weighs the risk, concern, or burden of extra care duties, then the intervention will be encouraged
	Benefits of exercise perceived by the carer or supporter for the person with dementia include; mood, behaviour, weight, flexibility, ageing, enjoyment of everyday life

For peer review only - http://bmjopen.bmj.com/site/about/guidelines.xhtml

List of Abbreviations

CMO: context-mechanism-outcome; CMOC: context-mechanism-outcome configuration; MRT: middle range theory; AD: Alzheimer's disease; MCI: mild cognitive impairment; MMSE: mini mental state examination; UK: United Kingdom; SDT: self-determination theory; AGT: achievement goal theory.

Declarations

Ethics approval

Not applicable.

Consent for publication or to participate

Not applicable.

Patient involvement

Patients were not involved.

Data sharing statement

The datasets used and/or analysed during the current study are available from the corresponding author on reasonable request.

Competing interests

The authors declare that they have no competing interests.

Funding statement

This paper presents independent research funded by the Alzheimer's Society, UK, with the Healthcare Management Trust through a Clinical Training Fellowship (grant number 206), and the

For peer review only - http://bmjopen.bmj.com/site/about/guidelines.xhtml

For peer review only

United Kingdom National Institute for Health Research under its Programme Development Grants for Applied Research funding scheme (RP-DG-0611-10013). The views expressed are those of the authors and not necessarily those of the National Health Service, the NIHR or the Department of Health.

Authors' contributions

VB completed the search, data extraction, analysis and synthesis of the review as part of her PhD (PhD in Rehabilitation and Ageing, School of Medicine, University of Nottingham). RHH, VHM, TM, and PAL supervised the development of the review and critically reviewed all text and theory development as part of the stakeholder group. JEH critically reviewed the findings against established motivational theories and assisted in content development. All authors contributed to the content of the review, read and approved the final manuscript.

Acknowledgements

We would like to thank Dr. Geoff Wong, the Nuffield Department of Primary Care Health Sciences, University of Oxford, for the invaluable help with developing the protocol and the expert knowledge of realist synthesis.

For peer review only

References

1. Sherrington, C., et al., *Exercise To Prevent Falls In Older Adults: An Updated Systematic Review And Meta-Analysis.* British Journal of Sports Medicine, 2016. **0**: p. 1-10.
2. Shaw, F.E., *Prevention of falls in older people with dementia.* Journal of Neural Transmission, 2007. **114**(10): p. 1259-64.
3. Lord, S.R., et al., *Falls In Older People: Risk Factors And Strategies For Prevention*. 2007: Cambridge University Press.
4. Shaw, F.E., et al., *Multifactorial Intervention After A Fall In Older People With Cognitive Impairment And Dementia Presenting To The Accident And Emergency*

For peer review only - http://bmjopen.bmj.com/site/about/guidelines.xhtml

Department: Randomised Controlled Trial. British Medical Journal 2003. **326**: p. 73-78.

5. Delbaere, K., et al., *Mild Cognitive Impairment As A Predictor Of Falls In Community-Dwelling Older People.* The American Journal of Geriatric Psychiatry, 2012. **20**(10): p. 845-853.
6. National Institute of Care and Excellence. *Clinical Guideline 161. Falls: Assessment And Prevention Of Falls In Older People* 2013 19/12/2014].
7. Pawson, R., et al., *Realist synthesis: an introduction.* Manchester: ESRC Research Methods Programme, University of Manchester, 2004.
8. Dalkin, S.M., et al., *What's in a mechanism? Development of a key concept in realist evaluation.* Implementation Science, 2015. **10**(49).
9. Jagosh, J., *The "Context + Mechanism" Association: Mastering a Key Heuristic in Realist Evaluation for Innovating Complex Programmes and Policy*, in *CECAN Webinar Series*. 2017: https://realistmethodology-cares.org/.
10. Pawson, R., *Evidence-based policy: A realist perspective*. 2006: Sage publications.
11. Pearson, M., et al., *Implementing health promotion in schools: protocol for a realist systematic review of research and experience in the United Kingdom (UK).* Syst Rev, 2012. **1**(48): p. 10.1186.
12. Booth, V., et al., *Understanding The Theoretical Underpinning Of The Exercise Component In A Fall Prevention Programme For Older Adults With Mild Dementia: A Realist Review Protocol.* Systematic Reviews, 2016. **5**(119).
13. Booth, V., *The influence of dementia on falls, gait and rehabilitation*. 2017, University of Nottingham.
14. Booth, V., et al., *Falls Prevention Interventions In Older Adults With Cognitive Impairment: A Systematic Review Of Reviews.* International Journal of Therapy & Rehabilitation, 2015. **22**(6).
15. Moore, G.F., et al., *Process evaluation of complex interventions: Medical Research Council guidance.* BMJ, 2015. **350**: p. h1258.
16. Pawson, R., et al., *Realist review–a new method of systematic review designed for complex policy interventions.* Journal of Health Services Research & Policy, 2005. **10**(suppl 1): p. 21-34.
17. Wong, G., et al., *RAMESES publication standards: realist syntheses.* BMC Medicine, 2013. **11**(1): p. 21.
18. Wong, G., et al., *Realist synthesis. RAMESES training materials.* London: The RAMESES Project, 2013.
19. Suttanon, P., et al., *Factors influencing commencement and adherence to a home-based balance exercise program for reducing risk of falls: Perceptions of people with Alzheimer's disease and their caregivers.* International Psychogeriatrics, 2012. **24**(7): p. 1172-1182.
20. Blane, D.N., et al., *Interventions targeted at primary care practitioners to improve the identification and referral of patients with co-morbid obesity: a realist review protocol.* Systematic Reviews, 2015. **4**(1): p. 1-8.
21. Wong, G., et al., *Interventions to improve antimicrobial prescribing of doctors in training: the IMPACT (IMProving Antimicrobial presCribing of doctors in Training) realist review.* BMJ Open, 2015. **5**: p. e009059.
22. Andrade, L.P., et al., *Benefits of multimodal exercise intervention for postural control and frontal cognitive functions in individuals with Alzheimer's disease: a*

For peer review only - http://bmjopen.bmj.com/site/about/guidelines.xhtml

controlled trial. Journal of the American Geriatrics Society, 2013. **61**(11): p. 1919-26.

23. Blankevoort, C.G., et al., *Review of effects of physical activity on strength, balance, mobility and ADL performance in elderly subjects with dementia (Structured abstract).* Dementia and Geriatric Cognitive Disorders, 2010. **30**(5): p. 392-402.

24. Burton, E., et al., *Effectiveness of exercise programs to reduce falls in older people with dementia living in the community: A systematic review and meta-analysis.* Clinical Interventions in Aging, 2015. **10**: p. 421-434.

25. Cedervall, Y. and A.C. Åberg, *Physical activity and implications on well-being in mild Alzheimer's disease: A qualitative case study on two men with dementia and their spouses.* Physiotherapy Theory and Practice, 2010. **26**(4): p. 226-239.

26. Cedervall, Y., S. Torres, and A.C. Åberg, *Maintaining well-being and selfhood through physical activity: experiences of people with mild Alzheimer's disease.* Aging & Mental Health, 2015. **19**(8): p. 679-688.

27. Chan, W.C., et al., *Efficacy of Physical Exercise in Preventing Falls in Older Adults With Cognitive Impairment: A Systematic Review and Meta-Analysis.* Journal of the American Medical Directors Association, 2015. **16**(2): p. 149-154.

28. Christofoletti, G., et al., *Effects of motor intervention in elderly patients with dementia: An analysis of randomized controlled trials.* Topics in Geriatric Rehabilitation, 2007. **23**(2): p. 149-154.

29. Frederiksen, K., et al., *Acceptability, safetyand feasibility of aerobic exercise in patients with mild-to-moderate Alzheimer's disease: A pilot study.* Alzheimer's and Dementia, 2012. **8**(4): p. 715-716.

30. Garuffi, M., et al., *Effects of resistance training on the performance of activities of daily living in patients with Alzheimer's disease.* Geriatrics & Gerontology International, 2013. **13**(2): p. 322-8.

31. Hauer, K., et al., *Effectiveness of physical training on motor performance and fall prevention in cognitively impaired older persons: a systematic review.* American Journal of Physical Medicine & Rehabilitation, 2006. **85**(10): p. 847-57.

32. Hauer, K., et al., *Physical training improves motor performance in people with dementia: A randomized controlled trial.* Journal of the American Geriatrics Society, 2012. **60**(1): p. 8-15.

33. Hernandez, S.S., et al., *Effects of physical activity on cognitive functions, balance and risk of falls in elderly patients with Alzheimer's dementia.* Revista Brasileira de Fisioterapia, 2010. **14**(1): p. 68-74.

34. Huger, D., et al., *Designing studies on the effectiveness of physical training in patients with cognitive impairment.* Zeitschrift fur Gerontologie und Geriatrie, 2009. **42**(1): p. 11-9.

35. Liu-Ambrose, T. and M.G. Donaldson, *Exercise and cognition in older adults: is there a role for resistance training programmes?* British Journal of Sports Medicine, 2009. **43**(1): p. 25-7.

36. Malthouse, R. and F. Fox, *Exploring experiences of physical activity among people with Alzheimer's disease and their spouse carers: a qualitative study.* Physiotherapy, 2014. **100**(2): p. 169-175.

37. Pitkala, K.H., et al., *Effects of the Finnish Alzheimer Disease Exercise Trial (FINALEX): A Randomized Controlled Trial.* Journal of the American Medical Association Internal Medicine, 2013. **173**(10): p. 894-901.

For peer review only - http://bmjopen.bmj.com/site/about/guidelines.xhtml

38. Ries, J.D., J.M. Drake, and C. Marino, *A small-group functional balance intervention for individuals with Alzheimer disease: A pilot study.* Journal of Neurologic Physical Therapy, 2010. **34**(1): p. 3-10.

39. Shimada, H., et al., *Effects of multicomponent exercise in older adults with mild cognitive impairment.* Alzheimer's and Dementia, 2014. **10**: p. 572.

40. Stubbs, B., et al., *What are the factors associated with physical activity (PA) participation in community dwelling adults with dementia? A systematic review of PA correlates.* Archives of Gerontology & Geriatrics, 2014. **59**(2): p. 195-203.

41. van Alphen, H.J., T. Hortobágyi, and M.J. van Heuvelen, *Barriers, motivators, and facilitators of physical activity in dementia patients: A systematic review.* Archives of Gerontology and Geriatrics, 2016.

42. Erickson, K.I., *Therapeutic effects of exercise on cognitive function.* Journal of the American Geriatrics Society, 2013. **61**(11): p. 2038-2039.

43. Kaite Shearn, Hilary Piercy, Peter Allmark and Julia Hirst, *Building 'down' from grand theory where programme theory is lacking*, in *2nd International Conference on Realist Evaluation and Synthesis: Advancing Principles, StrengtheningPractice*. 2016: Barbican, London.

44. Deci, E.L. and R. Ryan, *Intrinsic Motivation And Self-Determination In Human Behavior*. 1985: New York and London: Plenum.

45. Ng, J.Y., et al., *Self-determination theory applied to health contexts: A meta-analysis.* Perspectives on Psychological Science, 2012. **7**(4): p. 325-340.

46. Rohrbasser, A., S. Mickan, and J. Harris, *Exploring why quality circles work in primary health care: a realist review protocol.* Systematic Reviews, 2013. **2**(1): p. 1.

47. Hoffmann, T.C., et al., *Better Reporting Of Interventions: Template For Intervention Description And Replication (TIDieR) Checklist And Guide.* BMJ, 2014. **348**: p. 1687.

48. Harwood, R.H., et al., *A development study and randomised feasibility trial of a tailored intervention to improve activity and reduce falls in older adults with mild cognitive impairment and mild dementia.* Pilot and feasibility studies, 2018. **4**(1): p. 49.

49. Booth, V., et al., *Promoting activity, independence and stability in early dementia and mild cognitive impairment (PrAISED): development of an intervention for people with mild cognitive impairment and dementia.* Clinical Rehabilitation, 2018: p. 0269215518758149.

50. Lisa Burrows, Jos Latour, Ruth Garside, Abi Hall, Bridie Kent, *Understanding the impact of memory cafes for people with dementia and their carers: a realist review*, in *PROSPERO*. 2016, Centre for Reviews and Dissemination: University of York.: http://www.crd.york.ac.uk/PROSPERO/display_record.asp?ID=CRD42016035257.

51. Gill Windle, Samantha Gregory, Anna Goulding, Dave O'Brien, Andrew Newman, *Understanding the impact of visual arts interventions on the well-being and connectivity of people living with dementia: a realist review*, in *PROSPERO*. 2014, Centre for Reviews and Dissemination; University of York: http://www.crd.york.ac.uk/PROSPERO/display_record.asp?ID=CRD42014008702.

52. Frances Bunn, Claire Goodman, Katherine Froggatt, *The Namaste Care intervention to improve the quality of dying for people with advanced dementia living in care homes: a rapid realist review*, in *PROSPERO*. 2016, Centre for Reviews and Dissemination; University of York: http://www.crd.york.ac.uk/PROSPERO/display_record.asp?ID=CRD42016047512.

For peer review only - http://bmjopen.bmj.com/site/about/guidelines.xhtml

53. Handley, M., F. Bunn, and C. Goodman, *Interventions that support the creation of dementia friendly environments in health care: protocol for a realist review.* Systematic Reviews, 2015. **4**(1): p. 1.

54. Bunn, F., et al., *Managing diabetes in people with dementia: protocol for a realist review.* Systematic Reviews, 2016. **5**(1): p. 1.

55. Sims, S., G. Hewitt, and R. Harris, *Evidence of collaboration, pooling of resources, learning and role blurring in interprofessional healthcare teams: a realist synthesis.* Journal of Interprofessional Care, 2015. **29**(1): p. 20-25.

56. Sims, S., G. Hewitt, and R. Harris, *Evidence of a shared purpose, critical reflection, innovation and leadership in interprofessional healthcare teams: a realist synthesis.* Journal of Interprofessional Care, 2015. **29**(3): p. 209-215.

57. Greenhalgh, J., et al., *Functionality and feedback: a protocol for a realist synthesis of the collation, interpretation and utilisation of PROMs data to improve patient care.* BMJ Open, 2014. **4**(7): p. e005601.

58. Dalkin, S.M., et al., *Understanding integrated care pathways in palliative care using realist evaluation: a mixed methods study protocol.* BMJ Open, 2012. **2**(4): p. e001533.

59. Reddy, S., et al., *Evaluating impact of clinical guidelines using a realist evaluation framework.* Journal of Evaluation in Clinical Practice, 2015. **21**: p. 1114-1120.

60. Deci, E.L. and R.M. Ryan, *Intrinsic motivation and self-determination in human behavior.* . 1985: New York, NY: Plenum.

61. Deci, E.L. and R.M. Ryan, *The" what" and" why" of goal pursuits: Human needs and the self-determination of behavior.* Psychological inquiry, 2000. **11**(4): p. 227-268.

62. Murray, A., et al., *Effect of a Self-Determination Theory–Based Communication Skills Training Program on Physiotherapists' Psychological Support for Their Patients With Chronic Low Back Pain: A Randomized Controlled Trial.* Archives of Physical Medicine and Rehabilitation, 2015. **96**(5): p. 809-816.

63. Deci, E.L. and R.M. Ryan, *Facilitating optimal motivation and psychological well-being across life's domains.* Canadian Psychology/Psychologie canadienne, 2008. **49**(1): p. 14.

64. Teixeira, P.J., et al., *Exercise, physical activity, and self-determination theory: a systematic review.* International Journal of Behavioral Nutrition and Physical Activity, 2012. **9**(1): p. 78.

65. Kirkland, R.A., et al., *Basic psychological needs satisfaction, motivation, and exercise in older adults.* Activities, Adaptation & Aging, 2011. **35**(3): p. 181-196.

66. Ames, C., *Achievement goals and the classroom motivational climate.* Student perceptions in the classroom, 1992: p. 327-348.

67. Nicholls, J.G., *The competitive ethos and democratic education*. 1989: Harvard University Press.

68. Nicholls, J.G., *Achievement motivation: Conceptions of ability, subjective experience, task choice, and performance.* Psychological review, 1984. **91**(3): p. 328.

69. Duda, J.L. and H.K. Hall, *Achievement goal theory in sport: Recent extensions and future directions*, in *Handbook of research in sport psychology* R.N. Singer, H.A. Hausenblas, and C.M. Janelle, Editors. 2001, New York, NY: John Wiley and Sons, Inc. p. 417-434.

For peer review only - http://bmjopen.bmj.com/site/about/guidelines.xhtml

70. Standage, M. and J.L. Duda, *Motivational processes impacting physical activity engagement among older adults.*, in *Sport and exercise psychology: A developmental perspective* M. Weiss, Editor. 2003, Morgantown, WV: Fitness Information Technology. p. 365-381.

71. Gardner, B., G.-J. de Bruijn, and P. Lally, *A systematic review and meta-analysis of applications of the self-report habit index to nutrition and physical activity behaviours.* Annals of Behavioral Medicine, 2011. **42**(2): p. 174-187.

For peer review only

For peer review only - http://bmjopen.bmj.com/site/about/guidelines.xhtml

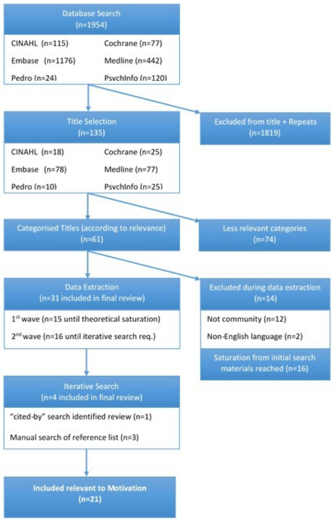

Figure 1: Flow diagram

338x190mm (96 x 96 DPI)

Supplementary Material

Supplementary Table 1: Questions for selection and quality appraisal

Selection Stage	Question
Screening Titles	Could this be about the strength and balance exercise component of falls rehabilitation in older adults with cognitive impairment in the community?
Screening Abstracts	Could this material provide useful information about completing the strength and balance exercise component of falls rehabilitation in older adults with cognitive impairment in the community?
Quality Appraisal of Articles	Is the material cohesive? Does it tell a comprehensive story or is there a juxtaposition of ideas or isolated statements?
	What is the value of the evidence?
	What is the material's position in relation to the programme theory and general topic area?

For peer review only

Supplementary Table 2: Quality appraisal of included studies

Reference	Study Design	Is the material cohesive?	What is the value?	What is the position in relation to the programme theories?	Broad/ Narrow	Thick/ Thin
**Suttanon (2012)**	Qualitative (interview)	Yes [Hill (2009)]	Considerable value	Adds and supports to many MRTs	Broad	Thick
**Cedervall (2015)**	Qualitative (interview)	Yes [Cedervall (2010)]	Considerable value	Adds to many MRTs	Broad	Thick
**van Alphen (2016)**	Literature (meta-synthesis)	Yes	Valuable	Supports and refutes the same MRT	Broad	Thick
**Cedervall (2010)**	Qualitative (case study)	Yes [Cedervall (2015)]	Valuable	Supports and refutes the some MRTs	Broad	Thick
**Malthouse (2014)**	Qualitative (interview)	Yes	Valuable	Adds to many MRTs	Broad	Thick
**De Andrade (2013)**	Non-randomised trial	Yes	Valuable	Adds to a few MRTs	Narrow	Thick
**Hernandez (2010)**	Non-randomised trial	Yes	Valuable	Adds to a few MRTs	Narrow	Thick
**Hauer (2012)**	Randomised trial	Yes	Valuable	Adds to a few MRTs	Narrow	Thick
**Pitkala (2013)**	Randomised trial	Yes	Valuable	Adds to few MRTs	Narrow	Thick
**Garuffi (2013)**	Non-randomised trial	Yes	Valuable	Adds to many MRTs	Broad	Thin
**Blankevoort (2010)**	Literature (systematic)	Yes	Valuable	Neither adds nor detracts from MRTs	Broad	Thin
**Burton (2015)**	Literature (meta-analysis)	Yes	Valuable	Limited in adding to MRTs development	Broad	Thin
**Ries (2010)**	Non-randomised trial	Yes	Valuable	Adds to many MRTs	Broad	Thin
**Stubbs (2014)**	Literature (systematic)	Yes	Valuable	Adds to many MRTs	Broad	Thin
**Huger (2009)**	Protocol	No	Limited value	Adds to few MRTs	Broad	Thin
**Frederiksen (2012)**	Abstract	Yes	Limited value	Relevant to few MRTs but cannot support or refute	Broad	Thin
**Hauer (2006)**	Literature (systematic)	Yes	Valuable	Adds to few MRTs	Narrow	Thin
**Chan (2015)**	Literature (meta-analysis)	Yes	Valuable	Adds to few MRTs	Narrow	Thin
**Christofoletti (2007)**	Literature (systematic)	Yes	Limited value	Adds to few MRTs	Narrow	Thin
**Liu-Ambrose (2009)**	Literature (non-systematic)	Yes	Limited value	Adds to few MRTs	Narrow	Thin
**Shimada (2014)**	Abstract	Yes	Limited value	Adds to few MRTs	Narrow	Thin

Reporting checklist for systematic review and meta-analysis.

Based on the PRISMA guidelines.

Instructions to authors

Complete this checklist by entering the page numbers from your manuscript where readers will find each of the items listed below.

Your article may not currently address all the items on the checklist. Please modify your text to include the missing information. If you are certain that an item does not apply, please write "n/a" and provide a short explanation.

Upload your completed checklist as an extra file when you submit to a journal.

In your methods section, say that you used the PRISMA reporting guidelines, and cite them as:

Moher D, Liberati A, Tetzlaff J, Altman DG, The PRISMA Group. Preferred Reporting Items for Systematic Reviews and Meta-Analyses: The PRISMA Statement

		Reporting Item	Page Number
	#1	Identify the report as a systematic review, meta-analysis, or both.	1
Structured summary	#2	Provide a structured summary including, as applicable: background; objectives; data sources; study eligibility criteria, participants, and interventions; study appraisal and synthesis methods; results; limitations; conclusions and implications of key findings; systematic review registration number	2-3
Rationale	#3	Describe the rationale for the review in the context of what is already known.	4-5
Objectives	#4	Provide an explicit statement of questions being addressed with reference to participants, interventions, comparisons, outcomes, and study design (PICOS).	5
Protocol and registration	#5	Indicate if a review protocol exists, if and where it can be accessed (e.g., Web address) and, if available, provide registration information including the registration number.	3 & 5

For peer review only

Eligibility criteria	#6	Specify study characteristics (e.g., PICOS, length of follow-up) and report characteristics (e.g., years considered, language, publication status) used as criteria for eligibility, giving rational	6-7
Information sources	#7	Describe all information sources in the search (e.g., databases with dates of coverage, contact with study authors to identify additional studies) and date last searched.	6-7
Search	#8	Present full electronic search strategy for at least one database, including any limits used, such that it could be repeated.	6-7
Study selection	#9	State the process for selecting studies (i.e., for screening, for determining eligibility, for inclusion in the systematic review, and, if applicable, for inclusion in the meta-analysis).	6-7
Data collection process	#10	Describe the method of data extraction from reports (e.g., piloted forms, independently by two reviewers) and any processes for obtaining and confirming data from investigators.	6-7
Data items	#11	List and define all variables for which data were sought (e.g., PICOS, funding sources), and any assumptions and simplifications made.	6-7
Risk of bias in individual studies	#12	Describe methods used for assessing risk of bias in individual studies (including specification of whether this was done at the study or outcome level, or both), and how this information is to be used in any data synthesis.	n/a
Summary measures	#13	State the principal summary measures (e.g., risk ratio, difference in means).	n/a
Planned methods of analyis	#14	Describe the methods of handling data and combining results of studies, if done, including measures of consistency (e.g., I2) for each meta-analysis.	6-7
Risk of bias across studies	#15	Specify any assessment of risk of bias that may affect the cumulative evidence (e.g., publication bias, selective reporting within studies).	n/a
Additional analyses	#16	Describe methods of additional analyses (e.g., sensitivity or subgroup analyses, meta-regression), if done, indicating which were pre-specified.	n/a
Study selection	#17	Give numbers of studies screened, assessed for eligibility, and	8

For peer review only

		included in the review, with reasons for exclusions at each stage, ideally with a flow diagram.	
Study characteristics	#18	For each study, present characteristics for which data were extracted (e.g., study size, PICOS, follow-up period) and provide the citation.	8
Risk of bias within studies	#19	Present data on risk of bias of each study and, if available, any outcome-level assessment (see Item 12).	8
Results of individual studies	#20	For all outcomes considered (benefits and harms), present, for each study: (a) simple summary data for each intervention group and (b) effect estimates and confidence intervals, ideally with a forest plot.	9-16
Synthesis of results	#21	Present the main results of the review. If meta-analyses are done, include for each, confidence intervals and measures of consistency.	9-16
Risk of bias across studies	#22	Present results of any assessment of risk of bias across studies (see Item 15).	n/a
Additional analysis	#23	Give results of additional analyses, if done (e.g., sensitivity or subgroup analyses, meta-regression [see Item 16]).	n/a
Summary of Evidence	#24	Summarize the main findings, including the strength of evidence for each main outcome; consider their relevance to key groups (e.g., health care providers, users, and policy makers	17
Limitations	#25	Discuss limitations at study and outcome level (e.g., risk of bias), and at review level (e.g., incomplete retrieval of identified research, reporting bias).	17-18
Conclusions	#26	Provide a general interpretation of the results in the context of other evidence, and implications for future research.	21
Funding	#27	Describe sources of funding or other support (e.g., supply of data) for the systematic review; role of funders for the systematic review.	23-24

The PRISMA checklist is distributed under the terms of the Creative Commons Attribution License CC-BY. This checklist was completed on 20. June 2018 using http://www.goodreports.org/, a tool made by the EQUATOR Network in collaboration with Penelope.ai

For peer review only

Motivation as a Mechanism Underpinning Exercise-based Falls Prevention Programmes for Older Adults with Cognitive Impairment: A Realist Review

Journal:	*BMJ Open*
Manuscript ID	bmjopen-2018-024982.R1
Article Type:	Research
Date Submitted by the Author:	20-Feb-2019
Complete List of Authors:	Booth, Vicky; University of Nottingham School of Medicine,<br>Harwood, Rowan; Health Care of Older People, Nottingham University Hospitals NHS Trust<br>Hancox, Jennie; University of Nottingham School of Medicine, Division of Rehabilitation and Ageing<br>Hood-Moore, Victoria; University of Nottingham School of Medicine, Division of Rehabilitation and Ageing<br>Masud, Tahir ; Nottingham University Hospital, Geriatric<br>Logan, Phillipa; The University of Nottingham, School of Medicine, Division of Rehabilitation and Ageing
**Primary Subject Heading**:	Rehabilitation medicine
Secondary Subject Heading:	Geriatric medicine, Research methods, Sports and exercise medicine
Keywords:	Dementia < NEUROLOGY, REHABILITATION MEDICINE, SPORTS MEDICINE

SCHOLARONE™
Manuscripts

Motivation as a Mechanism Underpinning Exercise-based Falls Prevention Programmes for Older Adults with Cognitive Impairment: A Realist Review

Authors

Vicky Booth, Rowan H Harwood, Jennie E Hancox, Victoria Hood-Moore, Tahir Masud, and Phillipa Logan.

Authors Address

Vicky Booth, School of Medicine and Health Sciences, University of Nottingham,
Victoria.Booth@nottingham.ac.uk (corresponding author)

Rowan H Harwood, School of Medicine and Health Sciences, University of Nottingham,
rowan.harwood@nottingham.ac.uk

Jennie E Hancox, University of Nottingham, mszjeh@nottingham.ac.uk

Victoria Hood-Moore, School of Medicine and Health Sciences, University of Nottingham,
mczvlh@nottingham.ac.uk

Tahir Masud, General Medicine, Nottingham University Hospitals NHS Trust,
Tahir.Masud@nuh.nhs.uk

Phillipa Logan, School of Medicine and Health Sciences, University of Nottingham,
Pip.Logan@nottingham.ac.uk

For peer review only - http://bmjopen.bmj.com/site/about/guidelines.xhtml

Abstract

Objectives

This review aimed to identify mechanisms underlying participation in falls prevention interventions, in older adults with cognitive impairment. In particular we studied the role of motivation.

Design

A realist review of the literature.

Data sources

EMBASE, MEDLINE, CINAHL, the Cochrane Library, PsycINFO, and PEDRO.

Eligibility criteria

Publications reporting exercise-based interventions for people with cognitive impairment, including dementia, living in the community.

Data extraction and synthesis

A "rough programme theory" (a preliminary model of how an intervention works) was developed, tested against findings from the published literature and refined. Data was collected according to elements of the programme theory and not isolated to outcomes. Motivation emerged as a key element, and was prioritised for further study.

Results

An individual will access mechanisms to support participation when they think that exercise will be beneficial to them. Supportive mechanisms include having a "gate-keeper", such as a carer or therapist, who shares responsibility for the perception of exercise as beneficial. Lack of access to support decreases adherence and participation in exercise. Motivational mechanisms were particularly relevant for older adults with mild to moderate dementia, where the exercise intervention was multicomponent, in a preferred setting, at the correct intensity and level of progression, correctly supported and considered, and flexibly delivered.

For peer review only

For peer review only - http://bmjopen.bmj.com/site/about/guidelines.xhtml

Conclusion

Motivation is a key element enabling participation in exercise-based interventions for people with cognitive impairment. Many of the mechanisms identified in this review have parallels in motivational theory. Clinically relevant recommendations were derived and will be used to further develop and test a motivationally considered exercise-based falls intervention for people with mild dementia.

Registration

PROSPERO CRD42015030169.

Keywords

Dementia; Cognitive Impairments; Exercise; Accidental Falls.

Word count

4699

Strengths and Limitations of this study

- Traditional systematic reviews report insufficient evidence to support falls prevention interventions amongst people with cognitive impairment.
- Realist review methodology explores what works for whom, in what circumstances and why (in contrast to systematic reviews which identify whether an intervention works or not), enabling exploration of contextual factors and underpinning mechanisms associated with exercise, and thus the necessary conditions for participation.
- We developed a programme theory explaining the role of motivation in exercise participation, and recommendations for clinicians to support exercise components of falls intervention programmes for older adults with mild cognitive impairment.
- Some elements of the programme theory were not well supported by evidence, limiting the depth and detail of the recommendations, in particular around the role of exercise specifically in falls prevention.

For peer review only - http://bmjopen.bmj.com/site/about/guidelines.xhtml

Introduction

Falls prevention represents a complex intervention due to the multifactorial causes of falls. There is robust evidence that falls risk can be reduced in the general older adult population [1], but for people with dementia the effectiveness of falls prevention is uncertain [2]. People with cognitive impairment have a high risk of falling [3-5], which frequently results in injury or hospital admission, but clinical guidelines cannot recommend evidence-based interventions [6].

Exercise, at the correct dose and intensity, reduces falls risk [1]. Motivation is defined as 'the energisation and direction of behavior' [7]. A key challenge is how to motivate older adults to achieve sufficient exercise participation and adherence to obtain such benefits, and this is especially so for people living with dementia. Exercise interventions with older adults with dementia have reported varying levels of adherence [8-11]. People with dementia undertake less physical activity compared to those without dementia [12]. A range of factors (e.g., problems with memory, executive function, carer burden, and co-morbidities) can influence exercise motivation [13]. However, people living with dementia populations vary according to level of impairment (mild to severe), diagnosis (e.g., Alzheimer's disease, frontotemporal dementia) and support (e.g., carer availability). Therefore, what is relevant to one individual might be different for another. Research is needed to explore the contextual factors and mechanisms associated with exercise engagement in older adults with dementia and to unravel some of the complexity as to what motivates whom, in what circumstances and why.

Realist synthesis is increasingly used for evaluating evidence for complex health and social interventions [14]. A realist review explores how underlying mechanisms (M) might be 'triggered' in the context (C) of a particular therapy in a particular population to produce an intended or unintended outcome (O). For example, someone with dementia who has fallen over before (C) may complete (O) an exercise programme because they are fearful (M) of falling over. Mechanisms are further

For peer review only - http://bmjopen.bmj.com/site/about/guidelines.xhtml

subdivided between resources, and responses [15]. Theory is generated and described through this Context-Mechanism-Outcome (CMO) heuristic [16]. CMO Configurations (CMOCs) can be linked, creating chains of possibilities and generating theories to explain why a particular outcome occurs with a specific intervention [17]. Interlinking CMOCs can be clustered together to form 'middle-range theories' (MRT), and in turn, a 'programme theory' or model of how an intervention works [14]. Realist methods encourage the incorporation of data from a range of sources, accommodating complexity that is inherent in health research [18].

Traditional systematic reviews examine the effectiveness of a defined intervention ('does it work?'), as opposed to exploring the underlying mechanisms, which, in theory, may be more generalizable when studying complex interventions in heterogeneous populations. A detailed rationale for completing a realist review in this field has been published [19, 20]. There are a limited studies in this field, which have used different research methods [21]. Developing a theoretical framework to rationalise and explain the key principles behind an intervention will aid its development and implementation [22].

The objective of this review was (i) to identify the underlying programme theory for participation in exercise-based falls prevention interventions in older adults with cognitive impairment, and (ii) to explore how and why that intervention reduces falls. The aim was to produce a list of recommendations that could be used clinically or to inform further intervention development.

Methods

Study Design

The review followed the stages identified by Pawson et al [23] including; i) articulating key rough programme theories to be explored, ii) searching for relevant evidence, iii) appraising the quality of

For peer review only - http://bmjopen.bmj.com/site/about/guidelines.xhtml

evidence, iv) extracting the data, and v) synthesising evidence. A detailed protocol has been published [19] and RAMESES guidance on publication of realist synthesis [24] reported (Supplementary Table 1).

Scoping

Initial scoping was based on prior knowledge of the falls prevention literature [21] and extensive clinical experience. The purpose was to clarify the aims of the review, develop an initial rough programme theory, and direct the search strategy for the main review.

Search processes

A phased literature search was conducted, initially in December 2015. An electronic search was completed of databases: EMBASE, MEDLINE, CINAHL, the Cochrane Library, PsycINFO, and PEDRO. Keywords and MESH headings were adapted according to the database used and included: *accidental falls, falls rehabilitation, falls prevention, exercise, dementia, cognitive impairment* (Supplementary Table 2).

The "*iterative and interactive*" [25] search process evolved during the review, utilising forward and backward citation checking and manual reference list search to find additional primary evidence that was required to refine a particular aspect of the programme theory. The forward-backward citation checking was completed using Google Scholar [26]. The reference list of a systematic review [18] was manually searched. During this second search phase material was specifically chosen for; i) focus on a population with dementia, ii) qualitative methods, and iii) reporting experiences of completing an exercise or physical-activity intervention. The search results were screened by the researcher (VB) who documented the number of articles retrieved during each search stage using EndNote reference management software.

For peer review only - http://bmjopen.bmj.com/site/about/guidelines.xhtml

Selection and appraisal of documents

Material was included regardless of study method (as is usual in realist reviews), but had to focus on an exercise intervention, be published in English and involve community-based participants or interventions. Reasons for exclusion were documented and a second researcher (PL or RH) consulted to aid decision making when required.

Titles and abstracts were screened by one researcher (VB) according to relevance of the material to the synthesis aims [27] (Supplementary Table 3). Quality appraisal of the articles was completed in accordance with standard practice of realist reviews [25]. Relevance and rigour were assessed using a series of judgements to define the quality (Supplementary Table 3).

The full-text for eligible studies was assessed for quality and extraction of data by one researcher (VB). A random sample of 10% of the materials was selected and assessed by a stakeholder group comprising rehabilitation and medical clinicians and academics.

Data extraction

Data were extracted based on relevance to the aims of the review and the rough programme theory. Data were sought that substantiated, refined or refuted the theories, and described contextual characteristics. Relevant material was highlighted, labelled, and recorded [28]. NVivo software and Excel was used to record and code the extracted data.

Analysis and synthesis process

Extracted material was coded as context, mechanism or outcome, and judgements regarding how this influenced the CMOCs recorded through annotations. Codes were initially allocated to each MRT within the rough programme theory, and as each article was processed, these codes were iteratively adapted according to the new material. Material that was relevant to more than one MRT were coded accordingly with links across theories.

For peer review only - http://bmjopen.bmj.com/site/about/guidelines.xhtml

Three waves of searching, analysis and synthesis occurred to direct the next stage of the review. Emerging findings were documented and then discussed with the stakeholder group.

Results

Document flow diagram

The initial search identified 1954 papers (Figure 1). Sixty-one papers were screened for inclusion. Sixteen papers from the initial search were not included as theoretical saturation had been reached (e.g., no new findings were emerging with the consideration of new papers). The iterative search identified a further four papers.

Figure 1: PRISMA Flow diagram for review documents.

Document characteristics

Twenty-one papers contributed data to the motivational mechanisms [13, 26, 29-47]. The papers varied in methodological design including; qualitative studies (n=4) [26, 32, 33, 43], literature reviews (n=8) [13, 30, 31, 34, 35, 38, 42, 47], randomised (n=2) [39, 44] and non-randomised trials (n=4) [29, 37, 40, 45], protocols (n=1) [41], and conference abstracts (n=2) [36, 46].

Contextual information, including the levels of cognitive impairment and the type, dose, and setting of the interventions, were summarised (Table 1 [20]).

Quality Appraisal

All of the papers were quality appraised and critiqued according to cohesion, value, position against other material and the rough programme theory (Supplementary Table 4).

For peer review only - http://bmjopen.bmj.com/site/about/guidelines.xhtml

Table 1: Characteristics of included studies with emphasis on the intervention.

Reference	Cognitive level	Type of intervention	Dose (*total hours*)	Setting
**Pitkala (2013)**	AD (67% mod/severe).	Intense, long-lasting physical exercise by dementia specialist physiotherapists in either home (HE) or group (GE) vs control (CG).	HE = 1 hr, 2 p/w, 12 mth (*104*); GE = 4 hr, 2 p/w, 12 mth (*416*).	Community, Finland.
**Shimada (2014)**	Amnesic MCI.	Multi-component group exercise by physiotherapists.	90 min, 2 p/w, (n=40 sessions) 10 mth (*130*).	Community, Japan.
**Hernandez (2010)**	AD (mild/mod).	Supervised group programme of regular physical activity.	1 hr, 3 p/w, 6 mth (*78*).	Community, Brazil.
**Hauer (2012)**	Dementia (mild/mod) (MMSE 21.7).	Progressive resistance and functional group training programme supervised by a qualified instructor.	2 hr, 2 p/w, 3 mth (*52*).	Community, Germany.
**de Andrade (2013)**	AD (mild/mod).	Aerobic, strength, flexibility, balance and cognitive exercises.	1 hr, 3 p/w, 16 wk (*48*).	Community, Brazil.
**Garuffi (2013)**	AD.	Resistance training.	1 hr, 3 p/w, 16 wk (*48*).	Community, Brazil.
**Huger (2009)**	Dementia (mild/mod, MMSE 17–26).	Progressive resistance and functional training including attention-depending motor-cognitive demands in small groups.	2 hr, 2 p/w, 12 wk (*48*).	Community, Germany
**Frederiksen (2012)**	AD (mild/mod).	Aerobic exercises (exercise machines) by physiotherapist.	1 hr, 3 p/w, 14 wk (*42*).	Community, Denmark.
**Ries (2010)**	AD (mean MMSE 23.2).	Balance exercise group with 2:1 supervision.	45 min, 2 p/w, 8 wk (*12*).	Community, USA.
**Suttanon (2012)**	AD (mild/mod).	Tailored home-based balance exercise by physiotherapist.	6 visits, 6 mth (*6*).	Community, Australia.
**Cedervall (2015)**	AD (mild).	Physical activity.	Range = "never" to 1 hr p/d.	Community, Sweden.
**Cedervall (2010)**	AD (mild).	Outdoor walking.	"Routine".	Community, Sweden.
**Malthouse (2014)**	AD (MMSE 18 to 21).	Physical activity.	"Routine".	Community, UK.
**Hauer (2006)**	Dementia (mod/severe)	Physical training.	Range=30–150 min, 2–7 p/w, 2–30 wk (*2-525*).	Mixed.
**Burton (2015)**	Dementia (MMSE 18.9).	Exercise or physical activity programme.	Range = 1-5 p/w, 3-12 mth (*13-260*).	Mixed.
**Blankevoort (2010)**	Dementia.	Physical activity.	Various.	Mixed.
**Chan (2015)**	Cognitive impairment.	Physical exercise.	Various.	Mixed.
**Stubbs (2014)**	Dementia.	Physical activity.	Various.	Community, mixed.
**Liu-Ambrose (2009)**	n/a.	Aerobic and resistance training.	2 p/w.	n/a
**van Alphen (2016)**	AD.	Physical activity.	n/a	Community, mixed.
**Christofoletti (2007)**	Dementia (mixed).	Motor intervention.	Various.	Mixed.

For peer review only

For peer review only - http://bmjopen.bmj.com/site/about/guidelines.xhtml

Motivation Programme Theory

The programme theory elaborated from the literature comprise eleven MRTs which explain how exercise is successfully undertaken. These included 'motivation', 'physiological response', 'enjoyment', 'empowered to achieve goals', 'fearful of negative consequences', 'influenced by social or cultural beliefs', 'depression', 'tailoring of intervention', 'energy', 'quality of life' and 'function in activities of daily living'. Motivation emerged as a mechanism from many of the included studies [13, 26, 29-47] and is described in detail. Two key mechanisms were shown to be operating; a) perceived benefit and b) support.

"Perception of benefit" response-mechanism

An older adult (C^{1}), with dementia (C^{2}), with the belief that exercise is advantageous (C^{3}) or a positive attitude to exercise (C^{4}), will perceive the benefit ($M^{response1}$) of completing (O^{1}) an exercise intervention ($M^{resource}$).

An older adult (C^{1}), with dementia (C^{2}), with either a carer or themselves having previous experience of being active and participating in exercise (C^{5}), and with a perception that exercise is beneficial (C^{6}), will feel encouraged ($M^{response2}$) to complete (O^{1}) an exercise intervention ($M^{resource}$).

An older adult (C^{1}), with dementia (C^{2}), who has the ability to comprehend (C^{7}) and identifies physical or functional changes (C^{8}) will perceive the benefit ($M^{response1}$) from completing (O^{1}) an exercise intervention ($M^{resource}$).

An older adult (C^{1}), with dementia (C^{2}), will perceive benefit ($M^{response1}$) from completing (O^{1}) an exercise intervention ($M^{resource}$) if they have an external supporter who either: understands the benefits of exercise; can prompt regarding the benefits of exercise; enables the person with dementia to compare themselves with others (C^{9}).

An older adult (C^{1}) with dementia (C^{2}) who has a carer who perceives the benefit the person with dementia doing exercise out-weighs the risk of doing exercise or the burden of extra care duties (C^{10}) will provide support ($M^{response3}$) for the participation (O^{1}) in an exercise programme ($M^{resource1}$) or routine physical activity ($M^{resource2}$).

An older adult (C^{1}) with dementia (C^{2}) who has a carer who perceives benefit on mood (C^{11}), behaviour (C^{12}), body weight (C^{13}), flexibility (C^{14}), ageing (C^{15}) and enjoyment of everyday life (C^{16}) for that older adult

For peer review only - http://bmjopen.bmj.com/site/about/guidelines.xhtml

doing exercise (C[17]) will provide support (M[response3]) for the participation (O[1]) in an exercise programme (M[resource1]) or routine physical activity (M[resource2]).

Box 1: Perception of benefit CMOCs.

The perception or feeling of benefit (Box 1 [20]) emerged from seven studies [13, 26, 30, 32, 33, 41, 43]. Perceiving the benefit of an exercise-based intervention could be either a response-mechanism or context, depending upon the individual and other context components [26]. The perception of benefit could be a response-mechanism that is operating when the person with cognitive impairment has "*prior experience of being active, participating in exercises, and perceiving benefits of general exercise*" (p1180, [26]) and is applicable to both the participant and carer [26, 32]. Understanding an individual's previous experience of exercise and their perceptions of it can allow tailoring of approaches [26]. Perception or belief in the benefits of exercise may also be a characteristic or feature (context) of the person with cognitive impairment or their carer, which encourages them to participate in the exercise intervention [17].

Recognition of improvements or changes in physiological-responses (for example in physical ability) reinforces an individual's perception of benefit [30]. Identification of benefit is important for both participation and the maintenance of an intervention [32, 33]. Huger et al [41] identified that persons with cognitive impairment can experience multiple problems which could include lack of comprehension (e.g., understanding the situation). This would influence ability to identify benefits from completing exercise, and whilst this statement seems negative, it could be interpreted as a context component for some individuals, rather than a general characteristic of all older adults with cognitive impairment.

Synergy is required between carers' understanding and support, ability to address barriers to exercise, and the participants' comprehension [13, 32]. Perceiving the health benefits for others also generates

For peer review only - http://bmjopen.bmj.com/site/about/guidelines.xhtml

support and encouragement that prompts participation (e.g., an individual walking his dog [32]). The feeling of encouragement could come from being able to compare themselves with others that have the same diagnosis, but only in the context where the older adult with dementia was doing well (e.g., being of good health, coping with dementia symptoms) [33].

The carer's perception and belief in the benefit of exercise must out-weigh the risk, care burden or adaption required to complete the exercise [32, 43]. Negative connotations associated with exercising (e.g., reminder of inability to do previously enjoyable activities), or adaptions or changes to routines or daily lives that are required to support the physical activity, are destructive to the perception of benefit for both the person providing the support and the person with dementia [32]. Concern can be both facilitator and barrier to engagement in exercise, requiring a judgement between not remaining mobile and healthy, against concern about getting lost or falling [43].

There was a range of perceived benefits from completing exercise which were not limited to health outcomes. A contentious benefit was an attempt to re-establish previous activities or the "person" that came before the dementia diagnosis or progression of dementia [32]. Cedervall and Aberg [32] reported this perception as coming from the person providing the support. The consideration of how exercise might influence dementia or benefit falls risk was not directly reported [43].

Support

An older adult (C^1) with dementia (C^2) who is supervised by trained personnel ($M^{resource1}$) who give clear/repeated instructions (C^3), optimally progress the exercises (C^4), provide the amount of supervision required by that individual and their needs (C^5), and understands the needs of persons with dementia (C^6) will feel supported ($M^{response1}$) to complete (O^1) an exercise programme ($M^{resource2}$).

An older adult (C^1) with dementia (C^2) who is being supported ($M^{response1}$) by a professional person ($M^{resource1}$) who can time-manage (C^7), is knowledgeable (C^8), firm but encouraging (C^9), is kind/friendly/supportive (C^{10}), who understands dementia (C^{11}) and can develop a rapport with the individual (C^{12}), will do an exercise programme (O^1).

For peer review only - http://bmjopen.bmj.com/site/about/guidelines.xhtml

An older adult (C^{1}) with dementia (C^{2}), who has a carer (C^{13}) who perceives a benefit from the older adult doing exercise (C^{14}) and can provide transport (C^{15}), a positive attitude (C^{16}), practical considerations (C^{17}), supportive strategies (C^{18}), and/or assistance (C^{19}) will feel supported ($M^{response1}$) to complete (O^{1}) an exercise programme ($M^{resource1}$) or routine physical activity ($M^{resource2}$).

An older adult (C^{1}) with more severe dementia (C^{20}) will require more support ($M^{response1}$) to successfully participate (O) in exercise programme ($M^{resource1}$) or routine physical activity ($M^{resource2}$).

An older adult (C^{1}) with dementia/AD (C^{2}) who has a carer (C^{13}) who receives information (C^{21}) and on-going support (C^{22}) from the therapist/staff ($M^{resource1}$) to enable them to support ($M^{response1}$) the participation/completion of an exercise programme of the person with dementia (O^{1}).

An older adult (C^{1}) with dementia (C^{2}) who wants to exercise in a group (C^{23}), will feel supported ($M^{response1}$) to complete (O^{1}) a group exercise programme ($M^{resouce1}$) or group physical activity ($M^{resource2}$).

An older adult (C^{1}) with dementia (C^{2}) who has a poorer ability to understand and learn new information (C^{24}), who has not exercised previously (C^{25}), has ill-health (C^{26}), or has regular holidays (C^{27}), will not access the required support needed to exercise ($M^{resource1}$) and therefore will not feel supported ($M^{response1}$) to complete an exercise programme(O^{1}).

Box 2: Support CMOCs.

Support was a mechanism of motivation (Box 2 [20]) reported in fourteen papers [13, 26, 30-36, 41, 43-45, 47]. Support could be provided through supervision [34, 41], practical measures [32], strategies such as making or maintaining routines [33] or through emotional support [33, 41]. There were many references to who provided the support and how it was given.

Supervision was discussed as a component of support [34]. Supervision by trained personnel "*met the special needs of persons with cognitive impairment*" (p153, [34]) by; giving clear and repeated instructions, optimally progressing the programme, and providing the amount of supervision required depending upon their ability to understand and learn new information [34]. Training instructors or supervisors provide more than just formal support during an intervention [41] and were influential in

For peer review only

For peer review only - http://bmjopen.bmj.com/site/about/guidelines.xhtml

the commencement, participation and maintenance of exercise [26]. Key characteristics of the professional person were identified [26]. An ability to "*understand my problem*" (p172, [43]) also emerged as important, particularly in regards to dementia. This facilitated rapport development between supporter and person with dementia, which included a relationship built on personal information [45] and trust [13].

Carer involvement was frequently reported and was an important component regarding the support they provided [47]. The role of the carer was described by Malthouse and Fox [43] as "*facilitators to activity*" and "*gate-keepers*". There were many ways in which carers provided support including the avoidance of stressful or negative situations [43], providing transport [29, 40], promoting a positive attitude [36], organising practical arrangements [32], employing specific strategies [33], providing additional assistance [47] and counteracting the "*loss of initiation and motivation*" [13] that were specific to that older adult with dementia and their situation. The promotion of exercise either in the practical or emotional sense, implies an underlying assumption that the activity is beneficial for the individual or themselves as a carer. However, this is a complex interaction illustrated by contradictory data. In some studies carers received encouragement, benefit or reduced distress from providing support for the person with cognitive impairment [35, 44]. In others they did not [13, 43, 44, 47], indicating the complexity of the caring role and the feelings associated with it.

Carers provided varying levels of support that were tailored to the individual [26, 32]. Carer involvement was integral to programme delivery in one study [26]. The carer and their support was more influential the more severe the cognitive impairment and may account for why people with severe dementia were still able to engage in interventions [47]. However, it was highlighted how complex the support component is, particularly as impairments progress [47]. Van Alphen et al [13] suggest that because persons with dementia require care and support, they are more influenced by support as a variable within an intervention. Carers themselves also required support, with information identified as a resource-mechanism to enable the support to take place [13, 26].

For peer review only - http://bmjopen.bmj.com/site/about/guidelines.xhtml

A group setting for the intervention provided support from both the trained staff and social aspects of the group [37, 40]. Individuals in the group contributed to the intervention, providing understanding of the issues and experiences of someone with dementia [13, 43]. Positive results from the social aspect of a group intervention were reported by some studies directly (e.g., through outcome measures) [26] and indirectly (e.g., through researcher opinion) [38]. However, this was not consistent across all studies [37]. Differences in participation and outcomes could be explained by the personality and preferences of the individuals. Some individuals had strong opinions on attending groups of people with dementia and this in itself will have influenced their participation [26, 39, 44].

Lack of support resulted in poor adherence or participation in exercise [31]. Contexts that contributed to poor participation included lack of previous exercise experience, ill-health and holidays [31]. Lack of support is also attributed to poor result in certain interventions trials [38] and as a barrier to physical activity [13].

Intervention Contextual Characteristics

An older adult (C^1) with mild to moderate dementia (C^2)

Interventions can be in either a home or group setting according to the preferences/wishes of the individual and their carer/spouse (C^3) (a home setting might be preferable for those wanting individual support from the intervention staff, or a group setting might be preferable for those wanting carer respite, or opportunities for social contact or engagement).

Interventions that are multicomponent combining physical (including strength/resistance, balance, endurance/mobility, aerobic) and cognitive exercises (C^4), at the correct intensity and level of progression (C^5), supported in the correct way by suitable staff and materials (interaction, communication and connection) (C^6), and with consideration for speed of initiation, length of intervention, encouragement of active lifestyle and enjoyment (C^7).

Intervention that is provided flexibly (C^8), for 6-12 months (C^9), 2-3 times a week (C^{10}), for minimum 15-20 minutes or whatever can be done or fit in with routine (C^{11}).

Box 3: Intervention Contextual CMOCs.

For peer review only - http://bmjopen.bmj.com/site/about/guidelines.xhtml

Many studies included participants with mild to moderate cognitive impairment (Box 3 [20]), with only two featuring moderate or severe dementia [38, 44]. Generalising across cognitive levels is not appropriate as improvements found from an intervention at one stage may not be found in another [38, 39]. A "*critical period*" for improvement within the cognitive impairment continuum was suggested [48].

Reports conflicted regarding whether a home [26] or group [44] setting was preferable. Characteristics of the different settings suited the preferences and wishes of different individuals and their situations. A solution where multiple settings (home and group) and locations (inside and outside) for the same intervention was suggested [13, 26].

Intensity and progression of exercise were important [29, 31, 45]. The influence of the intervention staff (interaction, communication and connection) [26, 43, 45] and the materials provided [26] were considered an asset of the intervention. Particular recommendations to consider the speed of initiation and length of intervention were identified [43]. Certain types of activity were more enjoyable for different individuals, as was the inclusion of certain types of exercise into the routine of daily life [13, 33, 43, 44].

The "dose" of an intervention is a combination of frequency, duration, and intensity. A range of doses was reported (Table 1). Overall, the optimal dose for an exercise intervention for persons with cognitive impairment has not been defined [37], is poorly understood [48], but is important [35]. The concept of "routine", both in content (such as a daily walk) and duration (such as fitting into daily life [26]) was highlighted [13, 32, 33, 43]. A flexible approach limited absences, particularly in consideration of the mood [43] or other health conditions [26] of the participant or their carer.

For peer review only - http://bmjopen.bmj.com/site/about/guidelines.xhtml

For peer review only

Discussion

Summary of findings

The review revealed motivation to be a core element of the programme theory underlying falls prevention interventions in older adults with cognitive impairment. Within the motivation component of the programme, two key mechanisms, perceived benefit and support, were shown to influence the extent to which an older adult with cognitive impairment is motivated to undertake an exercised-base intervention. When an older person with mild to moderate cognitive impairment believes that exercise will be beneficial they can utilise supportive mechanisms and contexts to complete an exercise programme. Support as a motivational mechanism requires a "*gate-keeper*", such as a therapist or carer, who shares or takes responsibility for the perception of exercise as beneficial, thereby enabling the person with dementia to access and participate in exercise programmes. A perception of benefit is both a mechanism and contextual feature within this programme theory. Lack of access to support had a detrimental effect on adherence and participation in exercise.

Strengths and limitations

This review progresses falls prevention research by using a novel approach. The main strength of this review is the successful completion of realist rationale in a historically positivist research field which prioritises causal probabilities over generalisability; an intervention may benefit a group on average, but we can be unsure if a given individual will benefit or be harmed. The realist review methodology was well-suited to the research question. Consideration of the mechanisms underpinning exercise-based interventions allowed development and extrapolation of the theoretical rationale. Exploring and documenting context components allows individualisation.

Transparency is encouraged in realist methods. The potential influence of the researcher in interpretation is acknowledged and, whilst being a potential source of bias, also assisted in the theory development and interpretations. Recognition of underlying or "*hidden*" mechanisms and

For peer review only

understanding of the CMOCs was strengthened by the experience of the main researcher (a physiotherapist) and the stakeholder group from their work with older people and falls prevention.

There are a number of limitations to this review. A micro (interpersonal) level [49] was the focus for the MRT's and overall programme theory, but the review did not consider meso (institutional) or macro (government and policy) levels of social structure [49]. The review did not base the rough programme theory on any overarching motivational theories (such as Self-Determination Theory [50, 51]). Theoretical frameworks are typically consulted to structure realist reviews (e.g., search strategies and data analysis) [49, 52].

The quality and content of the evidence available limited the review. Quantitative methods are more prevalent in research involving falls interventions with publications following specific reporting standards that does not encourage theoretical speculation. Information regarding participants and their influencing characteristics were rarely discussed. Greater contextual and resource information may be a product of recommendations for increased detail in reporting interventions (e.g., the TIDieR guidelines [53]).

None of the included studies provided any insight into potential CMO configurations linking exercise to falls. Included studies featured both exercise and physical activities interventions. Exercise is a valuable intervention to reduce falls risk, and yet these studies did not generate theory connecting falls prevention as an outcome or motivator to exercise for people with dementia. The results of the review reflect this and are therefore limited.

Only one researcher completed the screening and data extraction. Material relevant to the review may not have been identified. The literature search was conducted in 2015 and therefore further material may have been subsequently published. Further iterative searches and snow-ball searching was not completed in view of the restricted time and resources.

For peer review only

For peer review only - http://bmjopen.bmj.com/site/about/guidelines.xhtml

Future research directions

All of the materials included within the review described participants who had either completed regular physical-activity or the exercise-based intervention under study. The perspective of those not completing an exercise-based intervention must be considering for further programme theory refinement particularly considering the motivational mechanisms.

Further research could focus on the assessment and/or measurements of these mechanisms, for example by investigating the assessment of perceived benefit through use of measurements or scales.

The review process has clearly directed the need for a realist evaluation to test the refined programme theory. A realist evaluation could utilise data from an existing exercise-based intervention in people with mild dementia and cognitive impairment to assist participation and adherence [54, 55].

Comparison with existing literature

Realist reviews have previously been undertaken to explore issues involving people living with dementia [56-60] and aid explanation in other health systems and complex interventions [61-65].

These findings relate to a wider literature and existing theories of motivation. Many of the CMOs identified in this review have parallels with self-determination theory (SDT; [66, 67]). SDT is a theory of motivation which focuses on the mechanisms by which the social environment created by significant others (e.g., therapists or carers) influences individuals' motivation to engage in specific behaviours (e.g., exercise). Previous SDT research (e.g., Murray et al., [68]) has focused on the communication style utilised by healthcare professionals (e.g., what they say and how they say it) and the extent to which this satisfies participants' basic psychological needs (for competence, autonomy and relatedness [67]). This review has highlighted communication strategies (e.g., developing a rapport with the individual, being firm but encouraging, and promoting optimal progression) similar to those considered as need-supportive, and associated with participant completion of exercise

For peer review only - http://bmjopen.bmj.com/site/about/guidelines.xhtml

For peer review only

programmes. Thus, future intervention research within this population may want to consider drawing from SDT and training therapists and/or carers to adopt a need-supportive communication style.

Carer perception of benefits and support was found to be an important component of the programme theory. The 'perception of benefit' response-mechanism has parallels with the SDT mechanism of 'identified regulation'. Another key assumption of SDT is that there are qualitatively different reasons underlying behavioural engagement [69]. One of the more autonomous reasons for engaging in behaviour is identified regulation, which is when individuals engage in an activity because they identify with the benefits. Similarly to the CMOs presented within the 'perception of benefit' section of the results, research based on SDT has found that identifying with the benefits is an important mechanism mediating the relationship between the social context created by significant others (e.g., therapist or carers) and individuals' engagement in exercise behaviour [70]. Previous research has suggested that SDT is a suitable framework for investigating exercise engagement of older adults [71], however, SDT has not been applied in research exploring exercise participation in individuals with cognitive impairment or dementia. This review extends current knowledge by highlighting a potential limitation with the applicability of the SDT construct of identified regulation to all individuals with dementia as some may not have the psychological capability or capacity to comprehend (C^7) the benefits of taking part in the exercise programme.

Achievement Goal Theory (AGT) [72, 73] is another theory of motivation which has similarities to the findings of this review, and is also is conceptually related to SDT [74]. Similarly to SDT, AGT suggests that an important prerequisite for motivated behaviour is a desire to feel competent [75]. Results revealed comparison to others in a group setting as a motivating factor for older adults completing an exercise intervention but only when their performance is superior. AGT proposes that individuals can be more or less task- or ego-involved. Individuals who operate a more task-involved goal perspective perceive themselves as successful when they try their best and improve their own performance [76]. In contrast, more ego-involved individuals compare their performance to others and feel successful

For peer review only - http://bmjopen.bmj.com/site/about/guidelines.xhtml

only when their performance is superior [73]. Previous research suggests that although encouraging other-referenced comparisons may be a positive motivator in the short-term, in the long-term it can be associated with maladaptive outcomes such as lower levels of exercise participation [77]. Therefore, the inclusion of comparison against others in exercise interventions should be used with caution.

Making or maintaining routines was identified as a support mechanism associated with exercise programme completion. Similarly, a meta-analysis [78] found the creation of physical activity habits to offer a means to support maintenance of physical activity behaviours overtime. Thus, future research looking to support the development of routine exercise in older adults with dementia may want to consider psychological theory on non-conscious processes, such as habit-formation in order to support long-term exercise completion.

Conclusion and recommendations

Older adults with mild to moderate cognitive impairment experience falls. Interventions, such as exercise, should be considered a resource that can positively influence an outcome of preventing falls, when used in the right circumstances or contexts. This realist review highlighted that consideration of the circumstances and underlying mechanisms for exercise-based interventions are important and could lead to greater success for future research, the individuals involved and their support networks. Recommendations for what types of exercise-based interventions for people with dementia, under what circumstances would aid motivation are provided (Table 2 [20]).

For peer review only - http://bmjopen.bmj.com/site/about/guidelines.xhtml

Table 2: Clinically relevant recommendations from the review results

Focus	Recommendation
**Who**	Older adults with mild to moderate cognitive impairment
	If a person with dementia has the belief that exercise is advantageous, a positive attitude to exercise, the ability to understand the benefits of exercise or is able to identify the physical or functional changes from doing exercise, then they will perceive the benefit of doing exercise
	If a person with dementia perceives the benefit, they will participate in exercise-based intervention
**What**	Multicomponent exercise-based intervention that;<br>• combines physical (including strength/resistance, balance, endurance/mobility, aerobic) and cognitive exercises<br>• appropriately intensive and progressive<br>• supported by suitable staff (who can interact, communicate and connect) and materials<br>• considers speed of initiation, length of intervention, encouragement of active lifestyle and enjoyment<br>• is delivered in a flexible manner for at least 15-20 minutes (or whatever can become or fit in with routine) 2-3 times a week for 6-12 months<br>• can be delivered at home (for those wanting or needing 1:1 support from the intervention staff) or in a group (for those wanting carer respite, increase in habitual physical activity or socialising aspects)
**Circumstances**	Support can provide encouragement for completing an exercise-based intervention
	Sources of support can include but are not exclusively supplied by; trained intervention staff, carer, spouse, family member
	If support is being provided by trained intervention staff, then they should have professional competence including;<br>• time-management<br>• knowledgeable<br>• firm but encouraging<br>• kind, friendly and supportive<br>• understanding of the issues experienced by persons with dementia<br>• rapport development
	Trained intervention staff supporting an intervention should;<br>• provide clear and repeated instructions<br>• optimally progress the exercises<br>• provide the amount of supervision required by that individual and their needs<br>• understand the needs of persons with dementia
	If support is being provided by a carer, then the intervention should provide information and on-going support to enable them to continue
	Carers supporting an intervention should;<br>• perceive and understand the benefit of the person with dementia doing exercise<br>• provide transport or consider practical arrangements for access to the intervention<br>• have a belief in the benefit of exercise<br>• implement supportive strategies and/or assistance in the manner required by the person with dementia
	If the carers or supporters perception of the benefits of doing exercise out-weighs the risk, concern, or burden of extra care duties, then the intervention will be encouraged
	Benefits of exercise perceived by the carer or supporter for the person with dementia include; mood, behaviour, weight, flexibility, ageing, enjoyment of everyday life

For peer review only - http://bmjopen.bmj.com/site/about/guidelines.xhtml

List of Abbreviations & Figure Legends

CMO: context-mechanism-outcome; CMOC: context-mechanism-outcome configuration; MRT: middle range theory; AD: Alzheimer's disease; MCI: mild cognitive impairment; MMSE: mini mental state examination; UK: United Kingdom; SDT: self-determination theory; AGT: achievement goal theory; mod: moderate; n/a: not applicable; h: hour; p/w: per week; p/d: per day; mo: month; min: minutes.

Declarations

Ethics approval

Not applicable.

Consent for publication or to participate

Not applicable.

Patient involvement

Patients were not involved.

Data sharing statement

The datasets used and/or analysed during the current study are available from the corresponding author on reasonable request.

Competing interests

The authors declare that they have no competing interests.

For peer review only

For peer review only - http://bmjopen.bmj.com/site/about/guidelines.xhtml

Funding statement

This paper presents independent research funded by the Alzheimer's Society, UK, with the Healthcare Management Trust through a Clinical Training Fellowship (grant number 206), and the United Kingdom National Institute for Health Research under its Programme Development Grants for Applied Research funding scheme (RP-DG-0611-10013). The views expressed are those of the authors and not necessarily those of the National Health Service, the NIHR or the Department of Health.

Authors' contributions

VB completed the search, data extraction, analysis and synthesis of the review as part of her PhD (PhD in Rehabilitation and Ageing, School of Medicine, University of Nottingham). RHH, VHM, TM, and PAL supervised the development of the review and critically reviewed all text and theory development as part of the stakeholder group. JEH critically reviewed the findings against established motivational theories and assisted in content development. All authors contributed to the content of the review, read and approved the final manuscript.

Acknowledgements

We would like to thank Dr. Geoff Wong, the Nuffield Department of Primary Care Health Sciences, University of Oxford, for the invaluable help with developing the protocol and the expert knowledge of realist synthesis.

For peer review only

References

1. Sherrington, C., et al., *Exercise To Prevent Falls In Older Adults: An Updated Systematic Review And Meta-Analysis.* British Journal of Sports Medicine, 2016. **0**: p. 1-10.
2. Shaw, F.E., *Prevention of falls in older people with dementia.* Journal of Neural Transmission, 2007. **114**(10): p. 1259-64.

For peer review only - http://bmjopen.bmj.com/site/about/guidelines.xhtml

3. Lord, S.R., et al., *Falls In Older People: Risk Factors And Strategies For Prevention*. 2007: Cambridge University Press.

4. Shaw, F.E., et al., *Multifactorial Intervention After A Fall In Older People With Cognitive Impairment And Dementia Presenting To The Accident And Emergency Department: Randomised Controlled Trial.* British Medical Journal 2003. **326**: p. 73-78.

5. Delbaere, K., et al., *Mild Cognitive Impairment As A Predictor Of Falls In Community-Dwelling Older People.* The American Journal of Geriatric Psychiatry, 2012. **20**(10): p. 845-853.

6. National Institute of Care and Excellence. *Clinical Guideline 161. Falls: Assessment And Prevention Of Falls In Older People* 2013 19/12/2014].

7. Roberts, G.C., *Motivation in sport and exercise*. 1992: Human Kinetics Books Champaign, IL.

8. Van Der Wardt, V., et al., *Systematic Review Into Motivational Strategies That Support Adherence To Exercise For People With Mild Cognitive Impairment (MCI) Or Dementia.* European Geriatric Medicine, 2014. **5**(Supp. 1): p. S238.

9. Yu, F., *Improving recruitment, retention, and adherence to 6-month cycling in Alzheimer's disease.* Geriatric Nursing, 2013. **34**(3): p. 181-186.

10. Tak, E.C., et al., *Adherence to exercise programs and determinants of maintenance in older adults with mild cognitive impairment.* Journal of aging and physical activity, 2012. **20**(1): p. 32-46.

11. McCurry, S.M., et al., *Predictors of short-and long-term adherence to a daily walking program in persons with Alzheimer's disease.* American Journal of Alzheimer's Disease & Other Dementias®, 2010. **25**(6): p. 505-512.

12. Sabia, S., et al., *Physical activity, cognitive decline, and risk of dementia: 28 year follow-up of Whitehall II cohort study.* BMJ, 2017. **357**: p. j2709.

13. van Alphen, H.J., T. Hortobágyi, and M.J. van Heuvelen, *Barriers, motivators, and facilitators of physical activity in dementia patients: A systematic review.* Archives of Gerontology and Geriatrics, 2016.

14. Pawson, R., et al., *Realist synthesis: an introduction.* Manchester: ESRC Research Methods Programme, University of Manchester, 2004.

15. Dalkin, S.M., et al., *What's in a mechanism? Development of a key concept in realist evaluation.* Implementation Science, 2015. **10**(49).

16. Jagosh, J., *The "Context + Mechanism" Association: Mastering a Key Heuristic in Realist Evaluation for Innovating Complex Programmes and Policy*, in *CECAN Webinar Series*. 2017: https://realistmethodology-cares.org/.

17. Pawson, R., *Evidence-based policy: A realist perspective*. 2006: Sage publications.

18. Pearson, M., et al., *Implementing health promotion in schools: protocol for a realist systematic review of research and experience in the United Kingdom (UK).* Syst Rev, 2012. **1**(48): p. 10.1186.

19. Booth, V., et al., *Understanding The Theoretical Underpinning Of The Exercise Component In A Fall Prevention Programme For Older Adults With Mild Dementia: A Realist Review Protocol.* Systematic Reviews, 2016. **5**(119).

20. Booth, V., *The influence of dementia on falls, gait and rehabilitation*. 2017, University of Nottingham.

21. Booth, V., et al., *Falls Prevention Interventions In Older Adults With Cognitive Impairment: A Systematic Review Of Reviews.* International Journal of Therapy & Rehabilitation, 2015. **22**(6).

For peer review only - http://bmjopen.bmj.com/site/about/guidelines.xhtml

22. Moore, G.F., et al., *Process evaluation of complex interventions: Medical Research Council guidance.* BMJ, 2015. **350**: p. h1258.

23. Pawson, R., et al., *Realist review–a new method of systematic review designed for complex policy interventions.* Journal of Health Services Research & Policy, 2005. **10**(suppl 1): p. 21-34.

24. Wong, G., et al., *RAMESES publication standards: realist syntheses.* BMC Medicine, 2013. **11**(1): p. 21.

25. Wong, G., et al., *Realist synthesis. RAMESES training materials.* London: The RAMESES Project, 2013.

26. Suttanon, P., et al., *Factors influencing commencement and adherence to a home-based balance exercise program for reducing risk of falls: Perceptions of people with Alzheimer's disease and their caregivers.* International Psychogeriatrics, 2012. **24**(7): p. 1172-1182.

27. Blane, D.N., et al., *Interventions targeted at primary care practitioners to improve the identification and referral of patients with co-morbid obesity: a realist review protocol.* Systematic Reviews, 2015. **4**(1): p. 1-8.

28. Wong, G., et al., *Interventions to improve antimicrobial prescribing of doctors in training: the IMPACT (IMProving Antimicrobial presCribing of doctors in Training) realist review.* BMJ Open, 2015. **5**: p. e009059.

29. Andrade, L.P., et al., *Benefits of multimodal exercise intervention for postural control and frontal cognitive functions in individuals with Alzheimer's disease: a controlled trial.* Journal of the American Geriatrics Society, 2013. **61**(11): p. 1919-26.

30. Blankevoort, C.G., et al., *Review of effects of physical activity on strength, balance, mobility and ADL performance in elderly subjects with dementia (Structured abstract).* Dementia and Geriatric Cognitive Disorders, 2010. **30**(5): p. 392-402.

31. Burton, E., et al., *Effectiveness of exercise programs to reduce falls in older people with dementia living in the community: A systematic review and meta-analysis.* Clinical Interventions in Aging, 2015. **10**: p. 421-434.

32. Cedervall, Y. and A.C. Åberg, *Physical activity and implications on well-being in mild Alzheimer's disease: A qualitative case study on two men with dementia and their spouses.* Physiotherapy Theory and Practice, 2010. **26**(4): p. 226-239.

33. Cedervall, Y., S. Torres, and A.C. Åberg, *Maintaining well-being and selfhood through physical activity: experiences of people with mild Alzheimer's disease.* Aging & Mental Health, 2015. **19**(8): p. 679-688.

34. Chan, W.C., et al., *Efficacy of Physical Exercise in Preventing Falls in Older Adults With Cognitive Impairment: A Systematic Review and Meta-Analysis.* Journal of the American Medical Directors Association, 2015. **16**(2): p. 149-154.

35. Christofoletti, G., et al., *Effects of motor intervention in elderly patients with dementia: An analysis of randomized controlled trials.* Topics in Geriatric Rehabilitation, 2007. **23**(2): p. 149-154.

36. Frederiksen, K., et al., *Acceptability, safetyand feasibility of aerobic exercise in patients with mild-to-moderate Alzheimer's disease: A pilot study.* Alzheimer's and Dementia, 2012. **8**(4): p. 715-716.

37. Garuffi, M., et al., *Effects of resistance training on the performance of activities of daily living in patients with Alzheimer's disease.* Geriatrics & Gerontology International, 2013. **13**(2): p. 322-8.

For peer review only

For peer review only - http://bmjopen.bmj.com/site/about/guidelines.xhtml

38. Hauer, K., et al., *Effectiveness of physical training on motor performance and fall prevention in cognitively impaired older persons: a systematic review.* American Journal of Physical Medicine & Rehabilitation, 2006. **85**(10): p. 847-57.

39. Hauer, K., et al., *Physical training improves motor performance in people with dementia: A randomized controlled trial.* Journal of the American Geriatrics Society, 2012. **60**(1): p. 8-15.

40. Hernandez, S.S., et al., *Effects of physical activity on cognitive functions, balance and risk of falls in elderly patients with Alzheimer's dementia.* Revista Brasileira de Fisioterapia, 2010. **14**(1): p. 68-74.

41. Huger, D., et al., *Designing studies on the effectiveness of physical training in patients with cognitive impairment.* Zeitschrift fur Gerontologie und Geriatrie, 2009. **42**(1): p. 11-9.

42. Liu-Ambrose, T. and M.G. Donaldson, *Exercise and cognition in older adults: is there a role for resistance training programmes?* British Journal of Sports Medicine, 2009. **43**(1): p. 25-7.

43. Malthouse, R. and F. Fox, *Exploring experiences of physical activity among people with Alzheimer's disease and their spouse carers: a qualitative study.* Physiotherapy, 2014. **100**(2): p. 169-175.

44. Pitkala, K.H., et al., *Effects of the Finnish Alzheimer Disease Exercise Trial (FINALEX): A Randomized Controlled Trial.* Journal of the American Medical Association Internal Medicine, 2013. **173**(10): p. 894-901.

45. Ries, J.D., J.M. Drake, and C. Marino, *A small-group functional balance intervention for individuals with Alzheimer disease: A pilot study.* Journal of Neurologic Physical Therapy, 2010. **34**(1): p. 3-10.

46. Shimada, H., et al., *Effects of multicomponent exercise in older adults with mild cognitive impairment.* Alzheimer's and Dementia, 2014. **10**: p. 572.

47. Stubbs, B., et al., *What are the factors associated with physical activity (PA) participation in community dwelling adults with dementia? A systematic review of PA correlates.* Archives of Gerontology & Geriatrics, 2014. **59**(2): p. 195-203.

48. Erickson, K.I., *Therapeutic effects of exercise on cognitive function.* Journal of the American Geriatrics Society, 2013. **61**(11): p. 2038-2039.

49. Kaite Shearn, Hilary Piercy, Peter Allmark and Julia Hirst, *Building 'down' from grand theory where programme theory is lacking*, in *2nd International Conference on Realist Evaluation and Synthesis: Advancing Principles, StrengtheningPractice*. 2016: Barbican, London.

50. Deci, E.L. and R. Ryan, *Intrinsic Motivation And Self-Determination In Human Behavior*. 1985: New York and London: Plenum.

51. Ng, J.Y., et al., *Self-determination theory applied to health contexts: A meta-analysis.* Perspectives on Psychological Science, 2012. **7**(4): p. 325-340.

52. Rohrbasser, A., S. Mickan, and J. Harris, *Exploring why quality circles work in primary health care: a realist review protocol.* Systematic Reviews, 2013. **2**(1): p. 1.

53. Hoffmann, T.C., et al., *Better Reporting Of Interventions: Template For Intervention Description And Replication (TIDieR) Checklist And Guide.* BMJ, 2014. **348**: p. 1687.

54. Harwood, R.H., et al., *A development study and randomised feasibility trial of a tailored intervention to improve activity and reduce falls in older adults with mild cognitive impairment and mild dementia.* Pilot and feasibility studies, 2018. **4**(1): p. 49.

For peer review only - http://bmjopen.bmj.com/site/about/guidelines.xhtml

55. Booth, V., et al., *Promoting activity, independence and stability in early dementia and mild cognitive impairment (PrAISED): development of an intervention for people with mild cognitive impairment and dementia.* Clinical Rehabilitation, 2018: p. 0269215518758149.

56. Lisa Burrows, Jos Latour, Ruth Garside, Abi Hall, Bridie Kent, *Understanding the impact of memory cafes for people with dementia and their carers: a realist review*, in *PROSPERO*. 2016, Centre for Reviews and Dissemination: University of York.: http://www.crd.york.ac.uk/PROSPERO/display_record.asp?ID=CRD42016035257.

57. Gill Windle, Samantha Gregory, Anna Goulding, Dave O'Brien, Andrew Newman, *Understanding the impact of visual arts interventions on the well-being and connectivity of people living with dementia: a realist review*, in *PROSPERO*. 2014, Centre for Reviews and Dissemination; University of York: http://www.crd.york.ac.uk/PROSPERO/display_record.asp?ID=CRD42014008702.

58. Frances Bunn, Claire Goodman, Katherine Froggatt, *The Namaste Care intervention to improve the quality of dying for people with advanced dementia living in care homes: a rapid realist review*, in *PROSPERO*. 2016, Centre for Reviews and Dissemination; University of York: http://www.crd.york.ac.uk/PROSPERO/display_record.asp?ID=CRD42016047512.

59. Handley, M., F. Bunn, and C. Goodman, *Interventions that support the creation of dementia friendly environments in health care: protocol for a realist review.* Systematic Reviews, 2015. **4**(1): p. 1.

60. Bunn, F., et al., *Managing diabetes in people with dementia: protocol for a realist review.* Systematic Reviews, 2016. **5**(1): p. 1.

61. Sims, S., G. Hewitt, and R. Harris, *Evidence of collaboration, pooling of resources, learning and role blurring in interprofessional healthcare teams: a realist synthesis.* Journal of Interprofessional Care, 2015. **29**(1): p. 20-25.

62. Sims, S., G. Hewitt, and R. Harris, *Evidence of a shared purpose, critical reflection, innovation and leadership in interprofessional healthcare teams: a realist synthesis.* Journal of Interprofessional Care, 2015. **29**(3): p. 209-215.

63. Greenhalgh, J., et al., *Functionality and feedback: a protocol for a realist synthesis of the collation, interpretation and utilisation of PROMs data to improve patient care.* BMJ Open, 2014. **4**(7): p. e005601.

64. Dalkin, S.M., et al., *Understanding integrated care pathways in palliative care using realist evaluation: a mixed methods study protocol.* BMJ Open, 2012. **2**(4): p. e001533.

65. Reddy, S., et al., *Evaluating impact of clinical guidelines using a realist evaluation framework.* Journal of Evaluation in Clinical Practice, 2015. **21**: p. 1114-1120.

66. Deci, E.L. and R.M. Ryan, *Intrinsic motivation and self-determination in human behavior.* . 1985: New York, NY: Plenum.

67. Deci, E.L. and R.M. Ryan, *The" what" and" why" of goal pursuits: Human needs and the self-determination of behavior.* Psychological inquiry, 2000. **11**(4): p. 227-268.

68. Murray, A., et al., *Effect of a Self-Determination Theory–Based Communication Skills Training Program on Physiotherapists' Psychological Support for Their Patients With Chronic Low Back Pain: A Randomized Controlled Trial.* Archives of Physical Medicine and Rehabilitation, 2015. **96**(5): p. 809-816.

69. Deci, E.L. and R.M. Ryan, *Facilitating optimal motivation and psychological well-being across life's domains.* Canadian Psychology/Psychologie canadienne, 2008. **49**(1): p. 14.

For peer review only - http://bmjopen.bmj.com/site/about/guidelines.xhtml

70. Teixeira, P.J., et al., *Exercise, physical activity, and self-determination theory: a systematic review.* International Journal of Behavioral Nutrition and Physical Activity, 2012. **9**(1): p. 78.

71. Kirkland, R.A., et al., *Basic psychological needs satisfaction, motivation, and exercise in older adults.* Activities, Adaptation & Aging, 2011. **35**(3): p. 181-196.

72. Ames, C., *Achievement goals and the classroom motivational climate.* Student perceptions in the classroom, 1992: p. 327-348.

73. Nicholls, J.G., *The competitive ethos and democratic education*. 1989: Harvard University Press.

74. Ntoumanis, N., *Empirical links between achievement goal theory and self-determination theory in sport.* Journal of Sports Sciences, 2001. **19**(6): p. 397-409.

75. Nicholls, J.G., *Achievement motivation: Conceptions of ability, subjective experience, task choice, and performance.* Psychological review, 1984. **91**(3): p. 328.

76. Duda, J.L. and H.K. Hall, *Achievement goal theory in sport: Recent extensions and future directions*, in *Handbook of research in sport psychology* R.N. Singer, H.A. Hausenblas, and C.M. Janelle, Editors. 2001, New York, NY: John Wiley and Sons, Inc. p. 417-434.

77. Standage, M. and J.L. Duda, *Motivational processes impacting physical activity engagement among older adults.*, in *Sport and exercise psychology: A developmental perspective* M. Weiss, Editor. 2003, Morgantown, WV: Fitness Information Technology. p. 365-381.

78. Gardner, B., G.-J. de Bruijn, and P. Lally, *A systematic review and meta-analysis of applications of the self-report habit index to nutrition and physical activity behaviours.* Annals of Behavioral Medicine, 2011. **42**(2): p. 174-187.

For peer review only

For peer review only - http://bmjopen.bmj.com/site/about/guidelines.xhtml

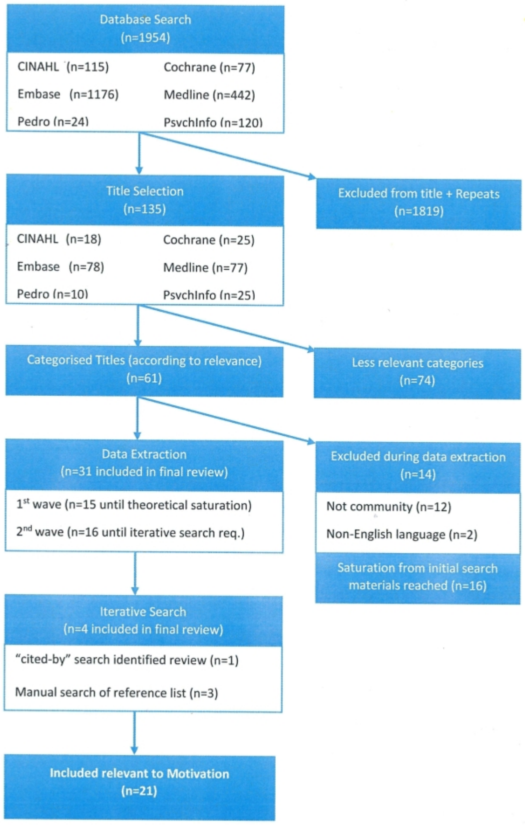

Figure 1

209x298mm (300 x 300 DPI)

Supplementary Material

Supplementary Table 1: RAMESES Realist Synthesis Checklist

TITLE		Reported in document Y/N/Unclear	Page(s) in document
1	In the title, identify the document as a realist synthesis or review	Y	1
ABSTRACT			
2	While acknowledging publication requirements and house style, abstracts should ideally contain brief details of: the study's background, review question or objectives; search strategy; methods of selection, appraisal, analysis and synthesis of sources; main results; and implications for practice.	Y	2-3
INTRODUCTION			
3 Rationale for review	Explain why the review is needed and what it is likely to contribute to existing understanding of the topic area.	Y	4-5
4 Objectives and focus of review	State the objective(s) of the review and/or the review question(s). Define and provide a rationale for the focus of the review.	Y	5
METHODS			
5 Changes in the review process	Any changes made to the review process that was initially planned should be briefly described and justified.	N	
6 Rationale for using realist synthesis	Explain why realist synthesis was considered the most appropriate method to use.	Y	4-5
7 Scoping the literature	Describe and justify the initial process of exploratory scoping of the literature.	Y	6
8 Searching processes	While considering specific requirements of the journal or other publication outlet, state and provide a rationale for how the iterative searching was done. Provide details on all the sources accessed for information in the review. Where searching in electronic databases has taken place, the details should include, for example, name of database, search terms, dates of coverage and date last searched. If individuals familiar with the relevant literature and/or topic area were contacted, indicate how they were identified and selected.	Y	6
9 Selection and appraisal of documents	Explain how judgements were made about including and excluding data from documents, and justify these.	Y	6-7
10 Data extraction	Describe and explain which data or information were extracted from the included documents and justify this selection.	Y	7
11 Analysis and synthesis processes	Describe the analysis and synthesis processes in detail. This section should include information on the constructs analyzed and describe the analytic process.	Y	7
RESULTS			
12 Document flow diagram	Provide details on the number of documents assessed for eligibility and included in the review with reasons for exclusion at each stage as well as an indication of	Y	8

For peer review only

	their source of origin (for example, from searching databases, reference lists and so on). You may consider using the example templates (which are likely to need modification to suit the data) that are provided.		
13 Document characteristics	Provide information on the characteristics of the documents included in the review.	Y	8-9
14 Main findings	Present the key findings with a specific focus on theory building and testing.	Y	10-16
DISCUSSION			
15 Summary of findings	Summarize the main findings, taking into account the review's objective(s), research question(s), focus and intended audience(s).	Y	17
16 Strengths, limitations and future research directions	Discuss both the strengths of the review and its limitations. These should include (but need not be restricted to) (a) consideration of all the steps in the review process and (b) comment on the overall strength of evidence supporting the explanatory insights which emerged.<br>The limitations identified may point to areas where further work is needed.	Y	17-19
17 Comparison with existing literature	Where applicable, compare and contrast the review's findings with the existing literature (for example, other reviews) on the same topic.	Y	19-21
18 Conclusion and recommendations	List the main implications of the findings and place these in the context of other relevant literature. If appropriate, offer recommendations for policy and practice.	Y	21
19 Funding	Provide details of funding source (if any) for the review, the role played by the funder (if any) and any conflicts of interests of the reviewers.	Y	23-24

For peer review only

Supplementary Table 2: Example search strategy

Cochrane search strategy<br><br>In Title, Abstract, Keywords<br><br>(accidental fall OR fall* OR fall risk OR postural balance) AND (mild cognitive impairment OR dementia OR Alzheimer disease OR cognit* impair*) AND (Aged OR elderly OR elder OR frail OR old* OR seniors OR geriatric OR older adult*) AND (Exercise OR "physical exercise" OR "exercise therapy" OR "physical activity" OR balance OR "resist* training" OR strength)

For peer review only

Supplementary Table 3: Questions for selection and quality appraisal

Selection Stage	Question
**Screening**	
Titles	Could this be about the strength and balance exercise component of falls rehabilitation in older adults with cognitive impairment in the community?
Abstracts	Could this material provide useful information about completing the strength and balance exercise component of falls rehabilitation in older adults with cognitive impairment in the community?
**Quality Appraisal of Articles**	
	Is the material cohesive? Does it tell a comprehensive story or is there a juxtaposition of ideas or isolated statements?
	What is the value of the evidence? Does it contribute to the topic area?
	What is the material's position in relation to the programme theory and general topic area?

For peer review only

Supplementary Table 4: Quality appraisal of included studies

Reference	Study Design	Is the material cohesive?	What is the value?	What is the position in relation to the programme theories?	Broad/ Narrow	Thick/ Thin
**Suttanon (2012)**	Qualitative (interview)	Yes [Hill (2009)]	Considerable value	Adds and supports to many MRTs	Broad	Thick
**Cedervall (2015)**	Qualitative (interview)	Yes [Cedervall (2010)]	Considerable value	Adds to many MRTs	Broad	Thick
**van Alphen (2016)**	Literature (meta-synthesis)	Yes	Valuable	Supports and refutes the same MRT	Broad	Thick
**Cedervall (2010)**	Qualitative (case study)	Yes [Cedervall (2015)]	Valuable	Supports and refutes the some MRTs	Broad	Thick
**Malthouse (2014)**	Qualitative (interview)	Yes	Valuable	Adds to many MRTs	Broad	Thick
**De Andrade (2013)**	Non-randomised trial	Yes	Valuable	Adds to a few MRTs	Narrow	Thick
**Hernandez (2010)**	Non-randomised trial	Yes	Valuable	Adds to a few MRTs	Narrow	Thick
**Hauer (2012)**	Randomised trial	Yes	Valuable	Adds to a few MRTs	Narrow	Thick
**Pitkala (2013)**	Randomised trial	Yes	Valuable	Adds to few MRTs	Narrow	Thick
**Garuffi (2013)**	Non-randomised trial	Yes	Valuable	Adds to many MRTs	Broad	Thin
**Blankevoort (2010)**	Literature (systematic)	Yes	Valuable	Neither adds nor detracts from MRTs	Broad	Thin
**Burton (2015)**	Literature (meta-analysis)	Yes	Valuable	Limited in adding to MRTs development	Broad	Thin
**Ries (2010)**	Non-randomised trial	Yes	Valuable	Adds to many MRTs	Broad	Thin
**Stubbs (2014)**	Literature (systematic)	Yes	Valuable	Adds to many MRTs	Broad	Thin
**Huger (2009)**	Protocol	No	Limited value	Adds to few MRTs	Broad	Thin
**Frederiksen (2012)**	Abstract	Yes	Limited value	Relevant to few MRTs but cannot support or refute	Broad	Thin
**Hauer (2006)**	Literature (systematic)	Yes	Valuable	Adds to few MRTs	Narrow	Thin
**Chan (2015)**	Literature (meta-analysis)	Yes	Valuable	Adds to few MRTs	Narrow	Thin
**Christofoletti (2007)**	Literature (systematic)	Yes	Limited value	Adds to few MRTs	Narrow	Thin
**Liu-Ambrose (2009)**	Literature (non-systematic)	Yes	Limited value	Adds to few MRTs	Narrow	Thin
**Shimada (2014)**	Abstract	Yes	Limited value	Adds to few MRTs	Narrow	Thin

For peer review only

BMJ Open

Motivation as a Mechanism Underpinning Exercise-based Falls Prevention Programmes for Older Adults with Cognitive Impairment: A Realist Review

Journal:	*BMJ Open*
Manuscript ID	bmjopen-2018-024982.R2
Article Type:	Research
Date Submitted by the Author:	13-Mar-2019
Complete List of Authors:	Booth, Vicky; University of Nottingham School of Medicine,<br>Harwood, Rowan; Health Care of Older People, Nottingham University Hospitals NHS Trust<br>Hancox, Jennie; University of Nottingham School of Medicine, Division of Rehabilitation and Ageing<br>Hood-Moore, Victoria; University of Nottingham School of Medicine, Division of Rehabilitation and Ageing<br>Masud, Tahir ; Nottingham University Hospital, Geriatric<br>Logan, Phillipa; The University of Nottingham, School of Medicine, Division of Rehabilitation and Ageing
**Primary Subject Heading**:	Rehabilitation medicine
Secondary Subject Heading:	Geriatric medicine, Research methods, Sports and exercise medicine
Keywords:	Dementia < NEUROLOGY, REHABILITATION MEDICINE, SPORTS MEDICINE

SCHOLARONE™
Manuscripts

Motivation as a Mechanism Underpinning Exercise-based Falls Prevention Programmes for Older Adults with Cognitive Impairment: A Realist Review

Authors

Vicky Booth, Rowan H Harwood, Jennie E Hancox, Victoria Hood-Moore, Tahir Masud, and Phillipa Logan.

Authors Address

Vicky Booth, School of Medicine and Health Sciences, University of Nottingham, Victoria.Booth@nottingham.ac.uk (corresponding author)

Rowan H Harwood, School of Medicine and Health Sciences, University of Nottingham, rowan.harwood@nottingham.ac.uk

Jennie E Hancox, University of Nottingham, mszjeh@nottingham.ac.uk

Victoria Hood-Moore, School of Medicine and Health Sciences, University of Nottingham, mczvlh@nottingham.ac.uk

Tahir Masud, General Medicine, Nottingham University Hospitals NHS Trust, Tahir.Masud@nuh.nhs.uk

Phillipa Logan, School of Medicine and Health Sciences, University of Nottingham, Pip.Logan@nottingham.ac.uk

For peer review only

For peer review only - http://bmjopen.bmj.com/site/about/guidelines.xhtml

Abstract

Objectives

This review aimed to identify mechanisms underlying participation in falls prevention interventions, in older adults with cognitive impairment. In particular we studied the role of motivation.

Design

A realist review of the literature.

Data sources

EMBASE, MEDLINE, CINAHL, the Cochrane Library, PsycINFO, and PEDRO.

Eligibility criteria

Publications reporting exercise-based interventions for people with cognitive impairment, including dementia, living in the community.

Data extraction and synthesis

A "rough programme theory" (a preliminary model of how an intervention works) was developed, tested against findings from the published literature and refined. Data was collected according to elements of the programme theory and not isolated to outcomes. Motivation emerged as a key element, and was prioritised for further study.

Results

An individual will access mechanisms to support participation when they think that exercise will be beneficial to them. Supportive mechanisms include having a "gate-keeper", such as a carer or therapist, who shares responsibility for the perception of exercise as beneficial. Lack of access to support decreases adherence and participation in exercise. Motivational mechanisms were particularly relevant for older adults with mild to moderate dementia, where the exercise intervention was multicomponent, in a preferred setting, at the correct intensity and level of progression, correctly supported and considered, and flexibly delivered.

For peer review only - http://bmjopen.bmj.com/site/about/guidelines.xhtml

Conclusion

Motivation is a key element enabling participation in exercise-based interventions for people with cognitive impairment. Many of the mechanisms identified in this review have parallels in motivational theory. Clinically relevant recommendations were derived and will be used to further develop and test a motivationally considered exercise-based falls intervention for people with mild dementia.

Registration

PROSPERO CRD42015030169.

Keywords

Dementia; Cognitive Impairments; Exercise; Accidental Falls.

Word count

4699

Strengths and Limitations of this study

- Traditional systematic reviews report insufficient evidence to support falls prevention interventions amongst people with cognitive impairment.
- Realist review methodology explores what works for whom, in what circumstances and why (in contrast to systematic reviews which identify whether an intervention works or not), enabling exploration of contextual factors and underpinning mechanisms associated with exercise, and thus the necessary conditions for participation.
- We developed a programme theory explaining the role of motivation in exercise participation, and recommendations for clinicians to support exercise components of falls intervention programmes for older adults with mild to moderate cognitive impairment.
- Some elements of the programme theory were not well supported by evidence, limiting the depth and detail of the recommendations, in particular around the role of exercise specifically in falls prevention.

For peer review only - http://bmjopen.bmj.com/site/about/guidelines.xhtml

- The cut-off date of the iterative searches and restricting to English language publications are limitations of this review, as further work may have been published that could have influenced the programme theory.

Introduction

Falls prevention represents a complex intervention due to the multifactorial causes of falls. There is robust evidence that some interventions can reduce falls risk in the general older adult population [1, 2], but for people with dementia the effectiveness of falls prevention is uncertain [3, 4]. People with cognitive impairment have a high risk of falling [5-7], which frequently results in injury or hospital admission, but clinical guidelines cannot recommend evidence-based interventions [8].

Exercise, at the correct dose and intensity, reduces falls risk [1]. Motivation is defined as 'the energisation and direction of behavior' [9]. A key challenge is how to motivate older adults to achieve sufficient exercise participation and adherence to obtain such benefits, and this is especially so for people living with dementia. Exercise interventions with older adults with dementia have reported varying levels of adherence [10-13]. People with dementia undertake less physical activity compared to those without dementia [14]. A range of factors (e.g., problems with memory, executive function, carer burden, and co-morbidities) can influence exercise motivation [15]. However, people living with dementia populations vary according to level of impairment (mild to severe), diagnosis (e.g., Alzheimer's disease, frontotemporal dementia) and support (e.g., carer availability). Therefore, what is relevant to one individual might be different for another. Research is needed to explore the contextual factors and mechanisms associated with exercise engagement in older adults with dementia and to unravel some of the complexity as to what motivates whom, in what circumstances and why.

Realist synthesis is increasingly used for evaluating evidence for complex health and social interventions [16]. A realist review explores how underlying mechanisms (M) might be 'triggered' in

For peer review only - http://bmjopen.bmj.com/site/about/guidelines.xhtml

the context (C) of a particular therapy in a particular population to produce an intended or unintended outcome (O). For example, someone with dementia who has fallen over before (C) may complete (O) an exercise programme because they are fearful (M) of falling over. Mechanisms are further subdivided between resources, and responses [17]. Theory is generated and described through this Context-Mechanism-Outcome (CMO) heuristic [18]. CMO Configurations (CMOCs) can be linked, creating chains of possibilities and generating theories to explain why a particular outcome occurs with a specific intervention [19]. Interlinking CMOCs can be clustered together to form 'middle-range theories' (MRT), and in turn, a 'programme theory' or model of how an intervention works [16]. Realist methods encourage the incorporation of data from a range of sources, accommodating complexity that is inherent in health research [20].

Traditional systematic reviews examine the effectiveness of a defined intervention ('does it work?'), as opposed to exploring the underlying mechanisms, which, in theory, may be more generalizable when studying complex interventions in heterogeneous populations. A detailed rationale for completing a realist review in this field has been published [21, 22]. There are a limited studies in this field, which have used different research methods [23]. Developing a theoretical framework to rationalise and explain the key principles behind an intervention will aid its development and implementation [24].

The objective of this review was (i) to identify the underlying programme theory for participation in exercise-based falls prevention interventions in older adults with cognitive impairment, and (ii) to explore how and why that intervention reduces falls. The aim was to produce a list of recommendations that could be used clinically or to inform further intervention development.

For peer review only - http://bmjopen.bmj.com/site/about/guidelines.xhtml

Methods

Study Design

The review followed the stages identified by Pawson et al [25] including; i) articulating key rough programme theories to be explored, ii) searching for relevant evidence, iii) appraising the quality of evidence, iv) extracting the data, and v) synthesising evidence. A detailed protocol has been published [21] and RAMESES guidance on publication of realist synthesis [26] reported (Supplementary Table 1).

Scoping

Initial scoping was based on prior knowledge of the falls prevention literature [23] and extensive clinical experience. The purpose was to clarify the aims of the review, develop an initial rough programme theory, and direct the search strategy for the main review.

Search processes

A phased literature search was conducted from December 2015 to June 2016. An electronic search was completed of databases: EMBASE, MEDLINE, CINAHL, the Cochrane Library, PsycINFO, and PEDRO. Keywords and MESH headings were adapted according to the database used and included: *accidental falls, falls rehabilitation, falls prevention, exercise, dementia, cognitive impairment* (Supplementary Table 2).

The *"iterative and interactive"* [27] search process evolved during the review, utilising forward and backward citation checking and manual reference list search to find additional primary evidence that was required to refine a particular aspect of the programme theory. The forward-backward citation checking was completed using Google Scholar [28]. The reference list of a systematic review [18] was manually searched. During this second search phase material was specifically chosen for; i) focus on a population with dementia, ii) qualitative methods, and iii) reporting experiences of completing an exercise or physical-activity intervention. The search results were screened by the researcher (VB)

For peer review only - http://bmjopen.bmj.com/site/about/guidelines.xhtml

who documented the number of articles retrieved during each search stage using EndNote reference management software.

Selection and appraisal of documents

Material was included regardless of study method (as is usual in realist reviews), but had to focus on an exercise intervention, be published in English and involve community-based participants or interventions. Reasons for exclusion were documented and a second researcher (PL or RH) consulted to aid decision making when required.

Titles and abstracts were screened by one researcher (VB) according to relevance of the material to the synthesis aims [29] (Supplementary Table 3). Full texts of the articles were appraised for quality in accordance with standard practice of realist reviews [27]. The relevance (*"does the research address the theory under test?"*, p7 [16]) and rigour (*"does the research support the conclusions drawn from it by the researchers?"*, p7 [16]) were assessed using a series of judgements to appraise the quality of the included studies (Supplementary Table 3).

The full-text for eligible studies was simultaneously assessed for quality and extraction of data by one researcher (VB). A random sample of 10% of the materials was selected and assessed by a stakeholder group comprising rehabilitation and medical clinicians and academics.

Data extraction

Data were extracted based on relevance to the aims of the review and the rough programme theory. Data were sought that substantiated, refined or refuted the theories, and described contextual characteristics. Relevant material was highlighted, labelled, and recorded [30]. NVivo software and Excel was used to record and code the extracted data.

For peer review only - http://bmjopen.bmj.com/site/about/guidelines.xhtml

For peer review only

Analysis and synthesis process

Extracted material was coded as context, mechanism or outcome, and judgements regarding how this influenced the CMOCs recorded through annotations. Codes were initially allocated to each MRT within the rough programme theory, and as each article was processed, these codes were iteratively adapted according to the new material. Material that was relevant to more than one MRT were coded accordingly with links across theories.

Three waves of searching, analysis and synthesis occurred to direct the next stage of the review. Emerging findings were documented and then discussed with the stakeholder group.

Patient and public involvement

Patients were not involved.

Results

Document flow diagram

The initial search identified 1954 papers (Figure 1). The full-text of sixty-one papers were eligible for screening. Sixteen papers from the initial search were not included as theoretical saturation had been reached (e.g., no new findings were emerging with the consideration of new papers). The iterative search identified a further four papers.

Figure 1: PRISMA Flow diagram for review documents.

Document characteristics

Twenty-one papers contributed data to the motivational mechanisms [4, 15, 28, 31-48]. The papers varied in methodological design including; qualitative studies (n=4) [28, 33, 34, 44], literature reviews

For peer review only - http://bmjopen.bmj.com/site/about/guidelines.xhtml

(n=8) [4, 15, 32, 35, 36, 39, 43, 48], randomised (n=2) [40, 45] and non-randomised trials (n=4) [31, 38, 41, 46], protocols (n=1) [42], and conference abstracts (n=2) [37, 47].

Contextual information, including the levels of cognitive impairment and the type, dose, and setting of the interventions, were summarised (Table 1 [22]).

Quality Appraisal

All of the papers were quality appraised and critiqued according to cohesion, value, position against other material and the rough programme theory (Supplementary Table 4).

For peer review only

For peer review only - http://bmjopen.bmj.com/site/about/guidelines.xhtml

Table 1: Characteristics of included studies with emphasis on the intervention.

Reference	Cognitive level	Type of intervention	Dose (*total hours*)	Setting
**Pitkala (2013)**	AD (67% mod/severe).	Intense, long-lasting physical exercise by dementia specialist physiotherapists in either home (HE) or group (GE) vs control (CG).	HE = 1 hr, 2 p/w, 12 mth (*104*); GE = 4 hr, 2 p/w, 12 mth (*416*).	Community, Finland.
**Shimada (2014)**	Amnesic MCI.	Multi-component group exercise by physiotherapists.	90 min, 2 p/w, (n=40 sessions) 10 mth (*130*).	Community, Japan.
**Hernandez (2010)**	AD (mild/mod).	Supervised group programme of regular physical activity.	1 hr, 3 p/w, 6 mth (*78*).	Community, Brazil.
**Hauer (2012)**	Dementia (mild/mod) (MMSE 21.7).	Progressive resistance and functional group training programme supervised by a qualified instructor.	2 hr, 2 p/w, 3 mth (*52*).	Community, Germany.
**de Andrade (2013)**	AD (mild/mod).	Aerobic, strength, flexibility, balance and cognitive exercises.	1 hr, 3 p/w, 16 wk (*48*).	Community, Brazil.
**Garuffi (2013)**	AD.	Resistance training.	1 hr, 3 p/w, 16 wk (*48*).	Community, Brazil.
**Huger (2009)**	Dementia (mild/mod, MMSE 17–26).	Progressive resistance and functional training including attention-depending motor-cognitive demands in small groups.	2 hr, 2 p/w, 12 wk (*48*).	Community, Germany
**Frederiksen (2012)**	AD (mild/mod).	Aerobic exercises (exercise machines) by physiotherapist.	1 hr, 3 p/w, 14 wk (*42*).	Community, Denmark.
**Ries (2010)**	AD (mean MMSE 23.2).	Balance exercise group with 2:1 supervision.	45 min, 2 p/w, 8 wk (*12*).	Community, USA.
**Suttanon (2012)**	AD (mild/mod).	Tailored home-based balance exercise by physiotherapist.	6 visits, 6 mth (*6*).	Community, Australia.
**Cedervall (2015)**	AD (mild).	Physical activity.	Range = “never” to 1 hr p/d.	Community, Sweden.
**Cedervall (2010)**	AD (mild).	Outdoor walking.	"Routine".	Community, Sweden.
**Malthouse (2014)**	AD (MMSE 18 to 21).	Physical activity.	"Routine".	Community, UK.
**Hauer (2006)**	Dementia (mod/severe)	Physical training.	Range=30–150 min, 2–7 p/w, 2–30 wk (*2-525*).	Mixed.
**Burton (2015)**	Dementia (MMSE 18.9).	Exercise or physical activity programme.	Range = 1-5 p/w, 3-12 mth (*13-260*).	Mixed.
**Blankevoort (2010)**	Dementia.	Physical activity.	Various.	Mixed.
**Chan (2015)**	Cognitive impairment.	Physical exercise.	Various.	Mixed.
**Stubbs (2014)**	Dementia.	Physical activity.	Various.	Community, mixed.
**Liu-Ambrose (2009)**	n/a.	Aerobic and resistance training.	2 p/w.	n/a
**van Alphen (2016)**	AD.	Physical activity.	n/a	Community, mixed.
**Christofoletti (2007)**	Dementia (mixed).	Motor intervention.	Various.	Mixed.

For peer review only

For peer review only - http://bmjopen.bmj.com/site/about/guidelines.xhtml

Motivation Programme Theory

The programme theory elaborated from the literature comprise eleven MRTs which explain how exercise is successfully undertaken. These included ‘motivation’, ‘physiological response’, ‘enjoyment’, ‘empowered to achieve goals’, ‘fearful of negative consequences’, ‘influenced by social or cultural beliefs’, ‘depression’, ‘tailoring of intervention’, ‘energy’, ‘quality of life’ and ‘function in activities of daily living’. Motivation emerged as a mechanism from many of the included studies [4, 15, 28, 31-48] and is described in detail. Two key mechanisms were shown to be operating; a) perceived benefit and b) support.

“Perception of benefit” response-mechanism

An older adult (C^1), with dementia (C^2), with the belief that exercise is advantageous (C^3) or a positive attitude to exercise (C^4), will perceive the benefit ($M^{response1}$) of completing (O^1) an exercise intervention ($M^{resource}$).

An older adult (C^1), with dementia (C^2), with either a carer or themselves having previous experience of being active and participating in exercise (C^5), and with a perception that exercise is beneficial (C^6), will feel encouraged ($M^{response2}$) to complete (O^1) an exercise intervention ($M^{resource}$).

An older adult (C^1), with dementia (C^2), who has the ability to comprehend (C^7) and identifies physical or functional changes (C^8) will perceive the benefit ($M^{response1}$) from completing (O^1) an exercise intervention ($M^{resource}$).

An older adult (C^1), with dementia (C^2), will perceive benefit ($M^{response1}$) from completing (O^1) an exercise intervention ($M^{resource}$) if they have an external supporter who either: understands the benefits of exercise; can prompt regarding the benefits of exercise; enables the person with dementia to compare themselves with others (C^9).

An older adult (C^1) with dementia (C^2) who has a carer who perceives the benefit the person with dementia doing exercise out-weighs the risk of doing exercise or the burden of extra care duties (C^{10}) will provide support ($M^{response3}$) for the participation (O^1) in an exercise programme ($M^{resource1}$) or routine physical activity ($M^{resource2}$).

An older adult (C^1) with dementia (C^2) who has a carer who perceives benefit on mood (C^{11}), behaviour (C^{12}), body weight (C^{13}), flexibility (C^{14}), ageing (C^{15}) and enjoyment of everyday life (C^{16}) for that older adult

For peer review only - http://bmjopen.bmj.com/site/about/guidelines.xhtml

doing exercise (C^{17}) will provide support ($M^{response3}$) for the participation (O^{1}) in an exercise programme ($M^{resource1}$) or routine physical activity ($M^{resource2}$).

Box 1: Perception of benefit CMOCs.

The perception or feeling of benefit (Box 1 [22]) emerged from seven studies [15, 28, 32-34, 42, 44]. Perceiving the benefit of an exercise-based intervention could be either a response-mechanism or context, depending upon the individual and other context components [28]. The perception of benefit could be a response-mechanism that is operating when the person with cognitive impairment has "*prior experience of being active, participating in exercises, and perceiving benefits of general exercise*" (p1180, [28]) and is applicable to both the participant and carer [28, 33]. Understanding an individual's previous experience of exercise and their perceptions of it can allow tailoring of approaches [28]. Perception or belief in the benefits of exercise may also be a characteristic or feature (context) of the person with cognitive impairment or their carer, which encourages them to participate in the exercise intervention [17].

Recognition of improvements or changes in physiological-responses (for example in physical ability) reinforces an individual's perception of benefit [32]. Identification of benefit is important for both participation and the maintenance of an intervention [33, 34]. Huger et al [42] identified that persons with cognitive impairment can experience multiple problems which could include lack of comprehension (e.g., understanding the situation). This would influence ability to identify benefits from completing exercise, and whilst this statement seems negative, it could be interpreted as a context component for some individuals, rather than a general characteristic of all older adults with cognitive impairment.

Synergy is required between carers' understanding and support, ability to address barriers to exercise, and the participants' comprehension [15, 33]. Perceiving the health benefits for others also generates

For peer review only

For peer review only - http://bmjopen.bmj.com/site/about/guidelines.xhtml

support and encouragement that prompts participation (e.g., an individual walking his dog [33]). The feeling of encouragement could come from being able to compare themselves with others that have the same diagnosis, but only in the context where the older adult with dementia was doing well (e.g., being of good health, coping with dementia symptoms) [34].

The carer's perception and belief in the benefit of exercise must out-weigh the risk, care burden or adaption required to complete the exercise [33, 44]. Negative connotations associated with exercising (e.g., reminder of inability to do previously enjoyable activities), or adaptions or changes to routines or daily lives that are required to support the physical activity, are destructive to the perception of benefit for both the person providing the support and the person with dementia [33]. Concern can be both facilitator and barrier to engagement in exercise, requiring a judgement between not remaining mobile and healthy, against concern about getting lost or falling [44].

There was a range of perceived benefits from completing exercise which were not limited to health outcomes. A contentious benefit was an attempt to re-establish previous activities or the "person" that came before the dementia diagnosis or progression of dementia [33]. Cedervall and Aberg [33] reported this perception as coming from the person providing the support. The consideration of how exercise might influence dementia or benefit falls risk was not directly reported [44].

Support

An older adult (C^{1}) with dementia (C^{2}) who is <u>supervised</u> by trained personnel ($M^{resource1}$) who give clear/repeated instructions (C^{3}), optimally progress the exercises (C^{4}), provide the amount of supervision required by that individual and their needs (C^{5}), and understands the needs of persons with dementia (C^{6}) will feel supported ($M^{response1}$) to complete (O^{1}) an exercise programme ($M^{resource2}$).

An older adult (C^{1}) with dementia (C^{2}) who is being supported ($M^{response1}$) by a <u>professional person</u> ($M^{resource1}$) who can time-manage (C^{7}), is knowledgeable (C^{8}), firm but encouraging (C^{9}), is kind/friendly/supportive (C^{10}), who understands dementia (C^{11}) and can develop a rapport with the individual (C^{12}), will do an exercise programme (O^{1}).

For peer review only

For peer review only - http://bmjopen.bmj.com/site/about/guidelines.xhtml

An older adult (C^{1}) with dementia (C^{2}), who has a carer (C^{13}) who perceives a benefit from the older adult doing exercise (C^{14}) and can provide transport (C^{15}), a positive attitude (C^{16}), practical considerations (C^{17}), supportive strategies (C^{18}), and/or assistance (C^{19}) will feel supported ($M^{response1}$) to complete (O^{1}) an exercise programme ($M^{resource1}$) or routine physical activity ($M^{resource2}$).

An older adult (C^{1}) with more severe dementia (C^{20}) will require more support ($M^{response1}$) to successfully participate (O) in exercise programme ($M^{resource1}$) or routine physical activity ($M^{resource2}$).

An older adult (C^{1}) with dementia/AD (C^{2}) who has a carer (C^{13}) who receives information (C^{21}) and on-going support (C^{22}) from the therapist/staff ($M^{resource1}$) to enable them to support ($M^{response1}$) the participation/completion of an exercise programme of the person with dementia (O^{1}).

An older adult (C^{1}) with dementia (C^{2}) who wants to exercise in a group (C^{23}), will feel supported ($M^{response1}$) to complete (O^{1}) a group exercise programme ($M^{resouce1}$) or group physical activity ($M^{resource2}$).

An older adult (C^{1}) with dementia (C^{2}) who has a poorer ability to understand and learn new information (C^{24}), who has not exercised previously (C^{25}), has ill-health (C^{26}), or has regular holidays (C^{27}), will not access the required support needed to exercise ($M^{resource1}$) and therefore will not feel supported ($M^{response1}$) to complete an exercise programme(O^{1}).

Box 2: Support CMOCs.

Support was a mechanism of motivation (Box 2 [22]) reported in fourteen papers [4, 15, 28, 32-37, 42, 44-46, 48]. Support could be provided through supervision [35, 42], practical measures [33], strategies such as making or maintaining routines [34] or through emotional support [34, 42]. There were many references to who provided the support and how it was given.

Supervision was discussed as a component of support [35]. Supervision by trained personnel "*met the special needs of persons with cognitive impairment*" (p153, [35]) by; giving clear and repeated instructions, optimally progressing the programme, and providing the amount of supervision required depending upon their ability to understand and learn new information [35]. Training instructors or supervisors provide more than just formal support during an intervention [42] and were influential in

For peer review only

For peer review only - http://bmjopen.bmj.com/site/about/guidelines.xhtml

the commencement, participation and maintenance of exercise [28]. Key characteristics of the professional person were identified [28]. An ability to "*understand my problem*" (p172, [44]) also emerged as important, particularly in regards to dementia. This facilitated rapport development between supporter and person with dementia, which included a relationship built on personal information [46] and trust [15].

Carer involvement was frequently reported and was an important component regarding the support they provided [48]. The role of the carer was described by Malthouse and Fox [44] as "*facilitators to activity*" and "*gate-keepers*". There were many ways in which carers provided support including the avoidance of stressful or negative situations [44], providing transport [31, 41], promoting a positive attitude [37], organising practical arrangements [33], employing specific strategies [34], providing additional assistance [48] and counteracting the "*loss of initiation and motivation*" [15] that were specific to that older adult with dementia and their situation. The promotion of exercise either in the practical or emotional sense, implies an underlying assumption that the activity is beneficial for the individual or themselves as a carer. However, this is a complex interaction illustrated by contradictory data. In some studies carers received encouragement, benefit or reduced distress from providing support for the person with cognitive impairment [36, 45]. In others they did not [15, 44, 45, 48], indicating the complexity of the caring role and the feelings associated with it.

Carers provided varying levels of support that were tailored to the individual [28, 33]. Carer involvement was integral to programme delivery in one study [28]. The carer and their support was more influential the more severe the cognitive impairment and may account for why people with severe dementia were still able to engage in interventions [48]. However, it was highlighted how complex the support component is, particularly as impairments progress [48]. Van Alphen et al [15] suggest that because persons with dementia require care and support, they are more influenced by support as a variable within an intervention. Carers themselves also required support, with information identified as a resource-mechanism to enable the support to take place [15, 28].

For peer review only - http://bmjopen.bmj.com/site/about/guidelines.xhtml

A group setting for the intervention provided support from both the trained staff and social aspects of the group [38, 41]. Individuals in the group contributed to the intervention, providing understanding of the issues and experiences of someone with dementia [15, 44]. Positive results from the social aspect of a group intervention were reported by some studies directly (e.g., through outcome measures) [28] and indirectly (e.g., through researcher opinion) [39]. However, this was not consistent across all studies [38]. Differences in participation and outcomes could be explained by the personality and preferences of the individuals. Some individuals had strong opinions on attending groups of people with dementia and this in itself will have influenced their participation [28, 40, 45].

Lack of support resulted in poor adherence or participation in exercise [4]. Contexts that contributed to poor participation included lack of previous exercise experience, ill-health and holidays [4]. Lack of support is also attributed to poor result in certain interventions trials [39] and as a barrier to physical activity [15].

Intervention Contextual Characteristics

An older adult (C^1) with mild to moderate dementia (C^2)

Interventions can be in either a home or group setting according to the preferences/wishes of the individual and their carer/spouse (C^3) (a home setting might be preferable for those wanting individual support from the intervention staff, or a group setting might be preferable for those wanting carer respite, or opportunities for social contact or engagement).

Interventions that are multicomponent combining physical (including strength/resistance, balance, endurance/mobility, aerobic) and cognitive exercises (C^4), at the correct intensity and level of progression (C^5), supported in the correct way by suitable staff and materials (interaction, communication and connection) (C^6), and with consideration for speed of initiation, length of intervention, encouragement of active lifestyle and enjoyment (C^7).

Intervention that is provided flexibly (C^8), for 6-12 months (C^9), 2-3 times a week (C^{10}), for minimum 15-20 minutes or whatever can be done or fit in with routine (C^{11}).

Box 3: Intervention Contextual CMOCs.

For peer review only - http://bmjopen.bmj.com/site/about/guidelines.xhtml

For peer review only

Many studies included participants with mild to moderate cognitive impairment (Box 3 [22]), with only two featuring moderate or severe dementia [39, 45]. Generalising across cognitive levels is not appropriate as improvements found from an intervention at one stage may not be found in another [39, 40]. A "*critical period*" for improvement within the cognitive impairment continuum was suggested [49].

Reports conflicted regarding whether a home [28] or group [45] setting was preferable. Characteristics of the different settings suited the preferences and wishes of different individuals and their situations. A solution where multiple settings (home and group) and locations (inside and outside) for the same intervention was suggested [15, 28].

Intensity and progression of exercise were important [4, 31, 46]. The influence of the intervention staff (interaction, communication and connection) [28, 44, 46] and the materials provided [28] were considered an asset of the intervention. Particular recommendations to consider the speed of initiation and length of intervention were identified [44]. Certain types of activity were more enjoyable for different individuals, as was the inclusion of certain types of exercise into the routine of daily life [15, 34, 44, 45].

The "dose" of an intervention is a combination of frequency, duration, and intensity. A range of doses was reported (Table 1). Overall, the optimal dose for an exercise intervention for persons with cognitive impairment has not been defined [38], is poorly understood [49], but is important [36]. The concept of "routine", both in content (such as a daily walk) and duration (such as fitting into daily life [28]) was highlighted [15, 33, 34, 44]. A flexible approach limited absences, particularly in consideration of the mood [44] or other health conditions [28] of the participant or their carer.

For peer review only - http://bmjopen.bmj.com/site/about/guidelines.xhtml

For peer review only

Discussion

Summary of findings

The review revealed motivation to be a core element of the programme theory underlying falls prevention interventions in older adults with cognitive impairment. Within the motivation component of the programme, two key mechanisms, perceived benefit and support, were shown to influence the extent to which an older adult with cognitive impairment is motivated to undertake an exercised-base intervention. When an older person with mild to moderate cognitive impairment believes that exercise will be beneficial they can utilise supportive mechanisms and contexts to complete an exercise programme. Support as a motivational mechanism requires a "*gate-keeper*", such as a therapist or carer, who shares or takes responsibility for the perception of exercise as beneficial, thereby enabling the person with dementia to access and participate in exercise programmes. A perception of benefit is both a mechanism and contextual feature within this programme theory. Lack of access to support had a detrimental effect on adherence and participation in exercise.

Strengths and limitations

This review progresses falls prevention research by using a novel approach. The main strength of this review is the successful completion of realist rationale in a historically positivist research field which prioritises causal probabilities over generalisability; an intervention may benefit a group on average, but we can be unsure if a given individual will benefit or be harmed. The realist review methodology was well-suited to the research question. Consideration of the mechanisms underpinning exercise-based interventions allowed development and extrapolation of the theoretical rationale. Exploring and documenting context components allows individualisation.

Transparency is encouraged in realist methods. The potential influence of the researcher in interpretation is acknowledged and, whilst being a potential source of bias, also assisted in the theory development and interpretations. Recognition of underlying or "*hidden*" mechanisms and

For peer review only - http://bmjopen.bmj.com/site/about/guidelines.xhtml

understanding of the CMOCs was strengthened by the experience of the main researcher (a physiotherapist) and the stakeholder group from their work with older people and falls prevention.

There are a number of limitations to this review. A micro (interpersonal) level [50] was the focus for the MRT's and overall programme theory, but the review did not consider meso (institutional) or macro (government and policy) levels of social structure [50]. The review did not base the rough programme theory on any overarching motivational theories (such as Self-Determination Theory [51, 52]). Theoretical frameworks are typically consulted to structure realist reviews (e.g., search strategies and data analysis) [50, 53].

The quality and content of the evidence available limited the review. Quantitative methods are more prevalent in research involving falls interventions with publications following specific reporting standards that does not encourage theoretical speculation. Information regarding participants and their influencing characteristics were rarely discussed. Greater contextual and resource information may be a product of recommendations for increased detail in reporting interventions (e.g., the TIDieR guidelines [54]).

None of the included studies provided any insight into potential CMO configurations linking exercise to falls. Included studies featured both exercise and physical activities interventions. Exercise is a valuable intervention to reduce falls risk, and yet these studies did not generate theory connecting falls prevention as an outcome or motivator to exercise for people with dementia. The results of the review reflect this and are therefore limited.

Only one researcher completed the screening and data extraction. Material relevant to the review may not have been identified. The literature search was conducted in 2015 and therefore further material may have been subsequently published. Further iterative searches and snow-ball searching

For peer review only - http://bmjopen.bmj.com/site/about/guidelines.xhtml

was not completed in view of the restricted time and resources. Only papers published in English were included.

Future research directions

All of the materials included within the review described participants who had either completed regular physical-activity or the exercise-based intervention under study. The perspective of those not completing an exercise-based intervention must be considering for further programme theory refinement particularly considering the motivational mechanisms.

Further research could focus on the assessment and/or measurements of these mechanisms, for example by investigating the assessment of perceived benefit through use of measurements or scales.

The review process has clearly directed the need for a realist evaluation to test the refined programme theory. A realist evaluation could utilise data from an existing exercise-based intervention in people with mild dementia and cognitive impairment to assist participation and adherence [55, 56].

Comparison with existing literature

Realist reviews have previously been undertaken to explore issues involving people living with dementia [57-61] and aid explanation in other health systems and complex interventions [62-66].

These findings relate to a wider literature and existing theories of motivation. Many of the CMOs identified in this review have parallels with self-determination theory (SDT; [67, 68]). SDT is a theory of motivation which focuses on the mechanisms by which the social environment created by significant others (e.g., therapists or carers) influences individuals' motivation to engage in specific behaviours (e.g., exercise). Previous SDT research (e.g., Murray et al., [69]) has focused on the communication style utilised by healthcare professionals (e.g., what they say and how they say it) and the extent to which this satisfies participants' basic psychological needs (for competence, autonomy

For peer review only - http://bmjopen.bmj.com/site/about/guidelines.xhtml

and relatedness [68]). This review has highlighted communication strategies (e.g., developing a rapport with the individual, being firm but encouraging, and promoting optimal progression) similar to those considered as need-supportive, and associated with participant completion of exercise programmes. Thus, future intervention research within this population may want to consider drawing from SDT and training therapists and/or carers to adopt a need-supportive communication style.

Carer perception of benefits and support was found to be an important component of the programme theory, which is also found in existing theories of motivation. The 'perception of benefit' response-mechanism has parallels with the SDT mechanism of 'identified regulation'. Another key assumption of SDT is that there are qualitatively different reasons underlying behavioural engagement [70]. One of the more autonomous reasons for engaging in behaviour is identified regulation, which is when individuals engage in an activity because they identify with the benefits. Similarly to the CMOs presented within the 'perception of benefit' section of the results, research based on SDT has found that identifying with the benefits is an important mechanism mediating the relationship between the social context created by significant others (e.g., therapist or carers) and individuals' engagement in exercise behaviour [71]. Previous research has suggested that SDT is a suitable framework for investigating exercise engagement of older adults [72], however, SDT has not been applied in research exploring exercise participation in individuals with cognitive impairment or dementia. This review extends current knowledge by highlighting a potential limitation with the applicability of the SDT construct of identified regulation to all individuals with dementia as some may not have the psychological capability or capacity to comprehend (C^7) the benefits of taking part in the exercise programme.

Achievement Goal Theory (AGT) [73, 74] is another theory of motivation which has similarities to the findings of this review, and is also is conceptually related to SDT [75]. Similarly to SDT, AGT suggests that an important prerequisite for motivated behaviour is a desire to feel competent [76]. Results revealed comparison to others in a group setting as a motivating factor for older adults completing an

For peer review only - http://bmjopen.bmj.com/site/about/guidelines.xhtml

exercise intervention but only when their performance is superior. AGT proposes that individuals can be more or less task- or ego-involved. Individuals who operate a more task-involved goal perspective perceive themselves as successful when they try their best and improve their own performance [77]. In contrast, more ego-involved individuals compare their performance to others and feel successful only when their performance is superior [74]. Previous research suggests that although encouraging other-referenced comparisons may be a positive motivator in the short-term, in the long-term it can be associated with maladaptive outcomes such as lower levels of exercise participation [78]. Therefore, the inclusion of comparison against others in exercise interventions should be used with caution.

Making or maintaining routines was identified as a support mechanism associated with exercise programme completion. Similarly, a meta-analysis [79] found the creation of physical activity habits to offer a means to support maintenance of physical activity behaviours overtime. Thus, future research looking to support the development of routine exercise in older adults with dementia may want to consider psychological theory on non-conscious processes, such as habit-formation in order to support long-term exercise completion.

Conclusion and recommendations

Older adults with mild to moderate cognitive impairment experience falls. Interventions, such as exercise, should be considered a resource that can positively influence an outcome of preventing falls, when used in the right circumstances or contexts. This realist review highlighted that consideration of the circumstances and underlying mechanisms for exercise-based interventions are important and could lead to greater success for future research, the individuals involved and their support networks. Recommendations for what types of exercise-based interventions for people with dementia, under what circumstances would aid motivation are provided (Table 2 [22]).

For peer review only - http://bmjopen.bmj.com/site/about/guidelines.xhtml

Table 2: Clinically relevant recommendations from the review results

Focus	Recommendation
**Who**	Older adults with mild to moderate cognitive impairment
	If a person with dementia has the belief that exercise is advantageous, a positive attitude to exercise, the ability to understand the benefits of exercise or is able to identify the physical or functional changes from doing exercise, then they will perceive the benefit of doing exercise
	If a person with dementia perceives the benefit, they will participate in exercise-based intervention
**What**	Multicomponent exercise-based intervention that;<br>• combines physical (including strength/resistance, balance, endurance/mobility, aerobic) and cognitive exercises<br>• appropriately intensive and progressive<br>• supported by suitable staff (who can interact, communicate and connect) and materials<br>• considers speed of initiation, length of intervention, encouragement of active lifestyle and enjoyment<br>• is delivered in a flexible manner for at least 15-20 minutes (or whatever can become or fit in with routine) 2-3 times a week for 6-12 months<br>• can be delivered at home (for those wanting or needing 1:1 support from the intervention staff) or in a group (for those wanting carer respite, increase in habitual physical activity or socialising aspects)
**Circumstances**	Support can provide encouragement for completing an exercise-based intervention
	Sources of support can include but are not exclusively supplied by; trained intervention staff, carer, spouse, family member
	If support is being provided by trained intervention staff, then they should have professional competence including;<br>• time-management<br>• knowledgeable<br>• firm but encouraging<br>• kind, friendly and supportive<br>• understanding of the issues experienced by persons with dementia<br>• rapport development
	Trained intervention staff supporting an intervention should;<br>• provide clear and repeated instructions<br>• optimally progress the exercises<br>• provide the amount of supervision required by that individual and their needs<br>• understand the needs of persons with dementia
	If support is being provided by a carer, then the intervention should provide information and on-going support to enable them to continue
	Carers supporting an intervention should;<br>• perceive and understand the benefit of the person with dementia doing exercise<br>• provide transport or consider practical arrangements for access to the intervention<br>• have a belief in the benefit of exercise<br>• implement supportive strategies and/or assistance in the manner required by the person with dementia
	If the carers or supporters perception of the benefits of doing exercise out-weighs the risk, concern, or burden of extra care duties, then the intervention will be encouraged
	Benefits of exercise perceived by the carer or supporter for the person with dementia include; mood, behaviour, weight, flexibility, ageing, enjoyment of everyday life

For peer review only - http://bmjopen.bmj.com/site/about/guidelines.xhtml

List of Abbreviations & Figure Legends

CMO: context-mechanism-outcome; CMOC: context-mechanism-outcome configuration; C^1: context numbered; $M^{resource1}$: mechanism resource numbered; $M^{response}$: mechanism response numbered; O^1: outcome numbered; MRT: middle range theory; AD: Alzheimer's disease; MCI: mild cognitive impairment; MMSE: mini mental state examination; UK: United Kingdom; SDT: self-determination theory; AGT: achievement goal theory; mod: moderate; n/a: not applicable; h: hour; p/w: per week; p/d: per day; mo: month; min: minutes.

Declarations

Ethics approval

Not applicable.

Consent for publication or to participate

Not applicable.

Data sharing statement

The datasets used and/or analysed during the current study are available from the corresponding author on reasonable request.

Competing interests

The authors declare that they have no competing interests.

Funding statement

This paper presents independent research funded by the Alzheimer's Society, UK, with the Healthcare Management Trust through a Clinical Training Fellowship (grant number 206), and the United Kingdom National Institute for Health Research under its Programme Development Grants for Applied Research

For peer review only

For peer review only - http://bmjopen.bmj.com/site/about/guidelines.xhtml

funding scheme (RP-DG-0611-10013). The views expressed are those of the authors and not necessarily those of the National Health Service, the NIHR or the Department of Health.

Authors' contributions

VB completed the search, data extraction, analysis and synthesis of the review as part of her PhD (PhD in Rehabilitation and Ageing, School of Medicine, University of Nottingham). RHH, VHM, TM, and PAL supervised the development of the review and critically reviewed all text and theory development as part of the stakeholder group. JEH critically reviewed the findings against established motivational theories and assisted in content development. All authors contributed to the content of the review, read and approved the final manuscript.

Acknowledgements

We would like to thank Dr. Geoff Wong, the Nuffield Department of Primary Care Health Sciences, University of Oxford, for the invaluable help with developing the protocol and the expert knowledge of realist synthesis.

References

1. Sherrington, C., et al., *Exercise To Prevent Falls In Older Adults: An Updated Systematic Review And Meta-Analysis.* British Journal of Sports Medicine, 2016. **0**: p. 1-10.
2. Gillespie, L.D., et al., *Interventions For Preventing Falls In Older People Living In The Community.* Cochrane Database Systematic Reviews, 2012. **9**.
3. Shaw, F.E., *Prevention of falls in older people with dementia.* Journal of Neural Transmission, 2007. **114**(10): p. 1259-64.
4. Burton, E., et al., *Effectiveness of exercise programs to reduce falls in older people with dementia living in the community: A systematic review and meta-analysis.* Clinical Interventions in Aging, 2015. **10**: p. 421-434.
5. Lord, S.R., et al., *Falls In Older People: Risk Factors And Strategies For Prevention*. 2007: Cambridge University Press.

For peer review only - http://bmjopen.bmj.com/site/about/guidelines.xhtml

6. Shaw, F.E., et al., *Multifactorial Intervention After A Fall In Older People With Cognitive Impairment And Dementia Presenting To The Accident And Emergency Department: Randomised Controlled Trial.* British Medical Journal 2003. **326**: p. 73-78.

7. Delbaere, K., et al., *Mild Cognitive Impairment As A Predictor Of Falls In Community-Dwelling Older People.* The American Journal of Geriatric Psychiatry, 2012. **20**(10): p. 845-853.

8. National Institute of Care and Excellence. *Clinical Guideline 161. Falls: Assessment And Prevention Of Falls In Older People* 2013 19/12/2014].

9. Roberts, G.C., *Motivation in sport and exercise*. 1992: Human Kinetics Books Champaign, IL.

10. Van Der Wardt, V., et al., *Systematic Review Into Motivational Strategies That Support Adherence To Exercise For People With Mild Cognitive Impairment (MCI) Or Dementia.* European Geriatric Medicine, 2014. **5**(Supp. 1): p. S238.

11. Yu, F., *Improving recruitment, retention, and adherence to 6-month cycling in Alzheimer's disease.* Geriatric Nursing, 2013. **34**(3): p. 181-186.

12. Tak, E.C., et al., *Adherence to exercise programs and determinants of maintenance in older adults with mild cognitive impairment.* Journal of aging and physical activity, 2012. **20**(1): p. 32-46.

13. McCurry, S.M., et al., *Predictors of short-and long-term adherence to a daily walking program in persons with Alzheimer's disease.* American Journal of Alzheimer's Disease & Other Dementias®, 2010. **25**(6): p. 505-512.

14. Sabia, S., et al., *Physical activity, cognitive decline, and risk of dementia: 28 year follow-up of Whitehall II cohort study.* BMJ, 2017. **357**: p. j2709.

15. van Alphen, H.J., T. Hortobágyi, and M.J. van Heuvelen, *Barriers, motivators, and facilitators of physical activity in dementia patients: A systematic review.* Archives of Gerontology and Geriatrics, 2016.

16. Pawson, R., et al., *Realist synthesis: an introduction.* Manchester: ESRC Research Methods Programme, University of Manchester, 2004.

17. Dalkin, S.M., et al., *What's in a mechanism? Development of a key concept in realist evaluation.* Implementation Science, 2015. **10**(49).

18. Jagosh, J., *The "Context + Mechanism" Association: Mastering a Key Heuristic in Realist Evaluation for Innovating Complex Programmes and Policy*, in *CECAN Webinar Series*. 2017: https://realistmethodology-cares.org/.

19. Pawson, R., *Evidence-based policy: A realist perspective*. 2006: Sage publications.

20. Pearson, M., et al., *Implementing health promotion in schools: protocol for a realist systematic review of research and experience in the United Kingdom (UK).* Syst Rev, 2012. **1**(48): p. 10.1186.

21. Booth, V., et al., *Understanding The Theoretical Underpinning Of The Exercise Component In A Fall Prevention Programme For Older Adults With Mild Dementia: A Realist Review Protocol.* Systematic Reviews, 2016. **5**(119).

22. Booth, V., *The influence of dementia on falls, gait and rehabilitation*. 2017, University of Nottingham.

23. Booth, V., et al., *Falls Prevention Interventions In Older Adults With Cognitive Impairment: A Systematic Review Of Reviews.* International Journal of Therapy & Rehabilitation, 2015. **22**(6).

24. Moore, G.F., et al., *Process evaluation of complex interventions: Medical Research Council guidance.* BMJ, 2015. **350**: p. h1258.

For peer review only - http://bmjopen.bmj.com/site/about/guidelines.xhtml

25. Pawson, R., et al., *Realist review–a new method of systematic review designed for complex policy interventions.* Journal of Health Services Research & Policy, 2005. **10**(suppl 1): p. 21-34.

26. Wong, G., et al., *RAMESES publication standards: realist syntheses.* BMC Medicine, 2013. **11**(1): p. 21.

27. Wong, G., et al., *Realist synthesis. RAMESES training materials.* London: The RAMESES Project, 2013.

28. Suttanon, P., et al., *Factors influencing commencement and adherence to a home-based balance exercise program for reducing risk of falls: Perceptions of people with Alzheimer's disease and their caregivers.* International Psychogeriatrics, 2012. **24**(7): p. 1172-1182.

29. Blane, D.N., et al., *Interventions targeted at primary care practitioners to improve the identification and referral of patients with co-morbid obesity: a realist review protocol.* Systematic Reviews, 2015. **4**(1): p. 1-8.

30. Wong, G., et al., *Interventions to improve antimicrobial prescribing of doctors in training: the IMPACT (IMProving Antimicrobial presCribing of doctors in Training) realist review.* BMJ Open, 2015. **5**: p. e009059.

31. Andrade, L.P., et al., *Benefits of multimodal exercise intervention for postural control and frontal cognitive functions in individuals with Alzheimer's disease: a controlled trial.* Journal of the American Geriatrics Society, 2013. **61**(11): p. 1919-26.

32. Blankevoort, C.G., et al., *Review of effects of physical activity on strength, balance, mobility and ADL performance in elderly subjects with dementia (Structured abstract).* Dementia and Geriatric Cognitive Disorders, 2010. **30**(5): p. 392-402.

33. Cedervall, Y. and A.C. Åberg, *Physical activity and implications on well-being in mild Alzheimer's disease: A qualitative case study on two men with dementia and their spouses.* Physiotherapy Theory and Practice, 2010. **26**(4): p. 226-239.

34. Cedervall, Y., S. Torres, and A.C. Åberg, *Maintaining well-being and selfhood through physical activity: experiences of people with mild Alzheimer's disease.* Aging & Mental Health, 2015. **19**(8): p. 679-688.

35. Chan, W.C., et al., *Efficacy of Physical Exercise in Preventing Falls in Older Adults With Cognitive Impairment: A Systematic Review and Meta-Analysis.* Journal of the American Medical Directors Association, 2015. **16**(2): p. 149-154.

36. Christofoletti, G., et al., *Effects of motor intervention in elderly patients with dementia: An analysis of randomized controlled trials.* Topics in Geriatric Rehabilitation, 2007. **23**(2): p. 149-154.

37. Frederiksen, K., et al., *Acceptability, safetyand feasibility of aerobic exercise in patients with mild-to-moderate Alzheimer's disease: A pilot study.* Alzheimer's and Dementia, 2012. **8**(4): p. 715-716.

38. Garuffi, M., et al., *Effects of resistance training on the performance of activities of daily living in patients with Alzheimer's disease.* Geriatrics & Gerontology International, 2013. **13**(2): p. 322-8.

39. Hauer, K., et al., *Effectiveness of physical training on motor performance and fall prevention in cognitively impaired older persons: a systematic review.* American Journal of Physical Medicine & Rehabilitation, 2006. **85**(10): p. 847-57.

40. Hauer, K., et al., *Physical training improves motor performance in people with dementia: A randomized controlled trial.* Journal of the American Geriatrics Society, 2012. **60**(1): p. 8-15.

For peer review only

For peer review only - http://bmjopen.bmj.com/site/about/guidelines.xhtml

41. Hernandez, S.S., et al., *Effects of physical activity on cognitive functions, balance and risk of falls in elderly patients with Alzheimer's dementia.* Revista Brasileira de Fisioterapia, 2010. **14**(1): p. 68-74.

42. Huger, D., et al., *Designing studies on the effectiveness of physical training in patients with cognitive impairment.* Zeitschrift fur Gerontologie und Geriatrie, 2009. **42**(1): p. 11-9.

43. Liu-Ambrose, T. and M.G. Donaldson, *Exercise and cognition in older adults: is there a role for resistance training programmes?* British Journal of Sports Medicine, 2009. **43**(1): p. 25-7.

44. Malthouse, R. and F. Fox, *Exploring experiences of physical activity among people with Alzheimer's disease and their spouse carers: a qualitative study.* Physiotherapy, 2014. **100**(2): p. 169-175.

45. Pitkala, K.H., et al., *Effects of the Finnish Alzheimer Disease Exercise Trial (FINALEX): A Randomized Controlled Trial.* Journal of the American Medical Association Internal Medicine, 2013. **173**(10): p. 894-901.

46. Ries, J.D., J.M. Drake, and C. Marino, *A small-group functional balance intervention for individuals with Alzheimer disease: A pilot study.* Journal of Neurologic Physical Therapy, 2010. **34**(1): p. 3-10.

47. Shimada, H., et al., *Effects of multicomponent exercise in older adults with mild cognitive impairment.* Alzheimer's and Dementia, 2014. **10**: p. 572.

48. Stubbs, B., et al., *What are the factors associated with physical activity (PA) participation in community dwelling adults with dementia? A systematic review of PA correlates.* Archives of Gerontology & Geriatrics, 2014. **59**(2): p. 195-203.

49. Erickson, K.I., *Therapeutic effects of exercise on cognitive function.* Journal of the American Geriatrics Society, 2013. **61**(11): p. 2038-2039.

50. Kaite Shearn, Hilary Piercy, Peter Allmark and Julia Hirst, *Building 'down' from grand theory where programme theory is lacking*, in *2nd International Conference on Realist Evaluation and Synthesis: Advancing Principles, StrengtheningPractice*. 2016: Barbican, London.

51. Deci, E.L. and R. Ryan, *Intrinsic Motivation And Self-Determination In Human Behavior*. 1985: New York and London: Plenum.

52. Ng, J.Y., et al., *Self-determination theory applied to health contexts: A meta-analysis.* Perspectives on Psychological Science, 2012. **7**(4): p. 325-340.

53. Rohrbasser, A., S. Mickan, and J. Harris, *Exploring why quality circles work in primary health care: a realist review protocol.* Systematic Reviews, 2013. **2**(1): p. 1.

54. Hoffmann, T.C., et al., *Better Reporting Of Interventions: Template For Intervention Description And Replication (TIDieR) Checklist And Guide.* BMJ, 2014. **348**: p. 1687.

55. Harwood, R.H., et al., *A development study and randomised feasibility trial of a tailored intervention to improve activity and reduce falls in older adults with mild cognitive impairment and mild dementia.* Pilot and feasibility studies, 2018. **4**(1): p. 49.

56. Booth, V., et al., *Promoting activity, independence and stability in early dementia and mild cognitive impairment (PrAISED): development of an intervention for people with mild cognitive impairment and dementia.* Clinical Rehabilitation, 2018: p. 0269215518758149.

57. Lisa Burrows, Jos Latour, Ruth Garside, Abi Hall, Bridie Kent, *Understanding the impact of memory cafes for people with dementia and their carers: a realist review,*

For peer review only - http://bmjopen.bmj.com/site/about/guidelines.xhtml

in *PROSPERO*. 2016, Centre for Reviews and Dissemination: University of York.: http://www.crd.york.ac.uk/PROSPERO/display_record.asp?ID=CRD42016035257.

58. Gill Windle, Samantha Gregory, Anna Goulding, Dave O'Brien, Andrew Newman, *Understanding the impact of visual arts interventions on the well-being and connectivity of people living with dementia: a realist review*, in *PROSPERO*. 2014, Centre for Reviews and Dissemination; University of York: http://www.crd.york.ac.uk/PROSPERO/display_record.asp?ID=CRD42014008702.

59. Frances Bunn, Claire Goodman, Katherine Froggatt, *The Namaste Care intervention to improve the quality of dying for people with advanced dementia living in care homes: a rapid realist review*, in *PROSPERO*. 2016, Centre for Reviews and Dissemination; University of York: http://www.crd.york.ac.uk/PROSPERO/display_record.asp?ID=CRD42016047512.

60. Handley, M., F. Bunn, and C. Goodman, *Interventions that support the creation of dementia friendly environments in health care: protocol for a realist review.* Systematic Reviews, 2015. **4**(1): p. 1.

61. Bunn, F., et al., *Managing diabetes in people with dementia: protocol for a realist review.* Systematic Reviews, 2016. **5**(1): p. 1.

62. Sims, S., G. Hewitt, and R. Harris, *Evidence of collaboration, pooling of resources, learning and role blurring in interprofessional healthcare teams: a realist synthesis.* Journal of Interprofessional Care, 2015. **29**(1): p. 20-25.

63. Sims, S., G. Hewitt, and R. Harris, *Evidence of a shared purpose, critical reflection, innovation and leadership in interprofessional healthcare teams: a realist synthesis.* Journal of Interprofessional Care, 2015. **29**(3): p. 209-215.

64. Greenhalgh, J., et al., *Functionality and feedback: a protocol for a realist synthesis of the collation, interpretation and utilisation of PROMs data to improve patient care.* BMJ Open, 2014. **4**(7): p. e005601.

65. Dalkin, S.M., et al., *Understanding integrated care pathways in palliative care using realist evaluation: a mixed methods study protocol.* BMJ Open, 2012. **2**(4): p. e001533.

66. Reddy, S., et al., *Evaluating impact of clinical guidelines using a realist evaluation framework.* Journal of Evaluation in Clinical Practice, 2015. **21**: p. 1114-1120.

67. Deci, E.L. and R.M. Ryan, *Intrinsic motivation and self-determination in human behavior.* . 1985: New York, NY: Plenum.

68. Deci, E.L. and R.M. Ryan, *The" what" and" why" of goal pursuits: Human needs and the self-determination of behavior.* Psychological inquiry, 2000. **11**(4): p. 227-268.

69. Murray, A., et al., *Effect of a Self-Determination Theory–Based Communication Skills Training Program on Physiotherapists' Psychological Support for Their Patients With Chronic Low Back Pain: A Randomized Controlled Trial.* Archives of Physical Medicine and Rehabilitation, 2015. **96**(5): p. 809-816.

70. Deci, E.L. and R.M. Ryan, *Facilitating optimal motivation and psychological well-being across life's domains.* Canadian Psychology/Psychologie canadienne, 2008. **49**(1): p. 14.

71. Teixeira, P.J., et al., *Exercise, physical activity, and self-determination theory: a systematic review.* International Journal of Behavioral Nutrition and Physical Activity, 2012. **9**(1): p. 78.

72. Kirkland, R.A., et al., *Basic psychological needs satisfaction, motivation, and exercise in older adults.* Activities, Adaptation & Aging, 2011. **35**(3): p. 181-196.

For peer review only - http://bmjopen.bmj.com/site/about/guidelines.xhtml

73. Ames, C., *Achievement goals and the classroom motivational climate.* Student perceptions in the classroom, 1992: p. 327-348.

74. Nicholls, J.G., *The competitive ethos and democratic education*. 1989: Harvard University Press.

75. Ntoumanis, N., *Empirical links between achievement goal theory and self-determination theory in sport.* Journal of Sports Sciences, 2001. **19**(6): p. 397-409.

76. Nicholls, J.G., *Achievement motivation: Conceptions of ability, subjective experience, task choice, and performance.* Psychological review, 1984. **91**(3): p. 328.

77. Duda, J.L. and H.K. Hall, *Achievement goal theory in sport: Recent extensions and future directions*, in *Handbook of research in sport psychology* R.N. Singer, H.A. Hausenblas, and C.M. Janelle, Editors. 2001, New York, NY: John Wiley and Sons, Inc. p. 417-434.

78. Standage, M. and J.L. Duda, *Motivational processes impacting physical activity engagement among older adults.*, in *Sport and exercise psychology: A developmental perspective* M. Weiss, Editor. 2003, Morgantown, WV: Fitness Information Technology. p. 365-381.

79. Gardner, B., G.-J. de Bruijn, and P. Lally, *A systematic review and meta-analysis of applications of the self-report habit index to nutrition and physical activity behaviours.* Annals of Behavioral Medicine, 2011. **42**(2): p. 174-187.

For peer review only

For peer review only - http://bmjopen.bmj.com/site/about/guidelines.xhtml

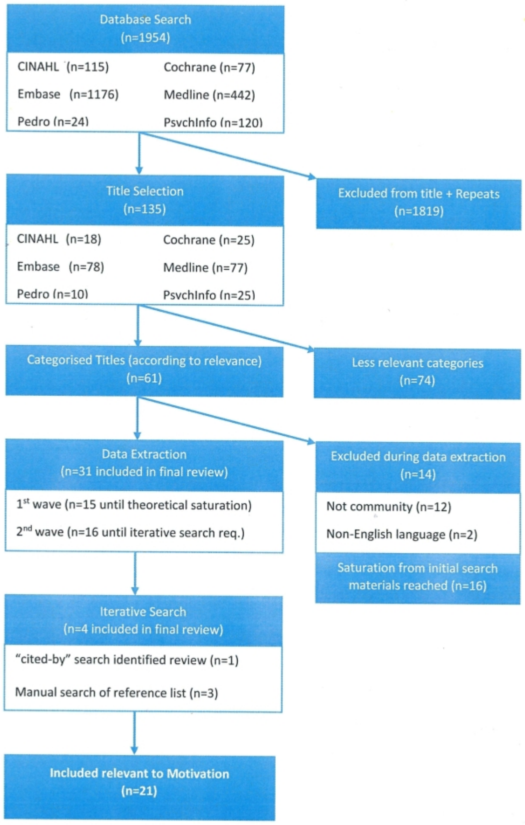

Figure 1

209x298mm (300 x 300 DPI)

Supplementary Material

Supplementary Table 1: RAMESES Realist Synthesis Checklist

TITLE		Reported in document Y/N/Unclear	Page(s) in document
1	In the title, identify the document as a realist synthesis or review	Y	1
ABSTRACT			
2	While acknowledging publication requirements and house style, abstracts should ideally contain brief details of: the study's background, review question or objectives; search strategy; methods of selection, appraisal, analysis and synthesis of sources; main results; and implications for practice.	Y	2-3
INTRODUCTION			
3 Rationale for review	Explain why the review is needed and what it is likely to contribute to existing understanding of the topic area.	Y	4-5
4 Objectives and focus of review	State the objective(s) of the review and/or the review question(s). Define and provide a rationale for the focus of the review.	Y	5
METHODS			
5 Changes in the review process	Any changes made to the review process that was initially planned should be briefly described and justified.	N	
6 Rationale for using realist synthesis	Explain why realist synthesis was considered the most appropriate method to use.	Y	4-5
7 Scoping the literature	Describe and justify the initial process of exploratory scoping of the literature.	Y	6
8 Searching processes	While considering specific requirements of the journal or other publication outlet, state and provide a rationale for how the iterative searching was done. Provide details on all the sources accessed for information in the review. Where searching in electronic databases has taken place, the details should include, for example, name of database, search terms, dates of coverage and date last searched. If individuals familiar with the relevant literature and/or topic area were contacted, indicate how they were identified and selected.	Y	6
9 Selection and appraisal of documents	Explain how judgements were made about including and excluding data from documents, and justify these.	Y	6-7
10 Data extraction	Describe and explain which data or information were extracted from the included documents and justify this selection.	Y	7
11 Analysis and synthesis processes	Describe the analysis and synthesis processes in detail. This section should include information on the constructs analyzed and describe the analytic process.	Y	7
RESULTS			
12 Document flow diagram	Provide details on the number of documents assessed for eligibility and included in the review with reasons for exclusion at each stage as well as an indication of	Y	8

For peer review only

	their source of origin (for example, from searching databases, reference lists and so on). You may consider using the example templates (which are likely to need modification to suit the data) that are provided.		
13 Document characteristics	Provide information on the characteristics of the documents included in the review.	Y	8-9
14 Main findings	Present the key findings with a specific focus on theory building and testing.	Y	10-16
DISCUSSION			
15 Summary of findings	Summarize the main findings, taking into account the review's objective(s), research question(s), focus and intended audience(s).	Y	17
16 Strengths, limitations and future research directions	Discuss both the strengths of the review and its limitations. These should include (but need not be restricted to) (a) consideration of all the steps in the review process and (b) comment on the overall strength of evidence supporting the explanatory insights which emerged.<br>The limitations identified may point to areas where further work is needed.	Y	17-19
17 Comparison with existing literature	Where applicable, compare and contrast the review's findings with the existing literature (for example, other reviews) on the same topic.	Y	19-21
18 Conclusion and recommendations	List the main implications of the findings and place these in the context of other relevant literature. If appropriate, offer recommendations for policy and practice.	Y	21
19 Funding	Provide details of funding source (if any) for the review, the role played by the funder (if any) and any conflicts of interests of the reviewers.	Y	23-24

For peer review only

Supplementary Table 2: Example search strategy

Cochrane search strategy<br><br>In Title, Abstract, Keywords<br><br>(accidental fall OR fall* OR fall risk OR postural balance) AND (mild cognitive impairment OR dementia OR Alzheimer disease OR cognit* impair*) AND (Aged OR elderly OR elder OR frail OR old* OR seniors OR geriatric OR older adult*) AND (Exercise OR "physical exercise" OR "exercise therapy" OR "physical activity" OR balance OR "resist* training" OR strength)

For peer review only

Supplementary Table 3: Questions for selection and quality appraisal

Selection Stage	Question
Screening	
Titles	Could this be about the strength and balance exercise component of falls rehabilitation in older adults with cognitive impairment in the community?
Abstracts	Could this material provide useful information about completing the strength and balance exercise component of falls rehabilitation in older adults with cognitive impairment in the community?
Quality Appraisal of Articles	
	Is the material cohesive? Does it tell a comprehensive story or is there a juxtaposition of ideas or isolated statements?
	What is the value of the evidence? Does it contribute to the topic area?
	What is the material's position in relation to the programme theory and general topic area?

For peer review only

Supplementary Table 4: Quality appraisal of included studies

Reference	Study Design	Is the material cohesive?	What is the value?	What is the position in relation to the programme theories?	Broad/ Narrow	Thick/ Thin
**Suttanon (2012)**	Qualitative (interview)	Yes [Hill (2009)]	Considerable value	Adds and supports to many MRTs	Broad	Thick
**Cedervall (2015)**	Qualitative (interview)	Yes [Cedervall (2010)]	Considerable value	Adds to many MRTs	Broad	Thick
**van Alphen (2016)**	Literature (meta-synthesis)	Yes	Valuable	Supports and refutes the same MRT	Broad	Thick
**Cedervall (2010)**	Qualitative (case study)	Yes [Cedervall (2015)]	Valuable	Supports and refutes the some MRTs	Broad	Thick
**Malthouse (2014)**	Qualitative (interview)	Yes	Valuable	Adds to many MRTs	Broad	Thick
**De Andrade (2013)**	Non-randomised trial	Yes	Valuable	Adds to a few MRTs	Narrow	Thick
**Hernandez (2010)**	Non-randomised trial	Yes	Valuable	Adds to a few MRTs	Narrow	Thick
**Hauer (2012)**	Randomised trial	Yes	Valuable	Adds to a few MRTs	Narrow	Thick
**Pitkala (2013)**	Randomised trial	Yes	Valuable	Adds to few MRTs	Narrow	Thick
**Garuffi (2013)**	Non-randomised trial	Yes	Valuable	Adds to many MRTs	Broad	Thin
**Blankevoort (2010)**	Literature (systematic)	Yes	Valuable	Neither adds nor detracts from MRTs	Broad	Thin
**Burton (2015)**	Literature (meta-analysis)	Yes	Valuable	Limited in adding to MRTs development	Broad	Thin
**Ries (2010)**	Non-randomised trial	Yes	Valuable	Adds to many MRTs	Broad	Thin
**Stubbs (2014)**	Literature (systematic)	Yes	Valuable	Adds to many MRTs	Broad	Thin
**Huger (2009)**	Protocol	No	Limited value	Adds to few MRTs	Broad	Thin
**Frederiksen (2012)**	Abstract	Yes	Limited value	Relevant to few MRTs but cannot support or refute	Broad	Thin
**Hauer (2006)**	Literature (systematic)	Yes	Valuable	Adds to few MRTs	Narrow	Thin
**Chan (2015)**	Literature (meta-analysis)	Yes	Valuable	Adds to few MRTs	Narrow	Thin
**Christofoletti (2007)**	Literature (systematic)	Yes	Limited value	Adds to few MRTs	Narrow	Thin
**Liu-Ambrose (2009)**	Literature (non-systematic)	Yes	Limited value	Adds to few MRTs	Narrow	Thin
**Shimada (2014)**	Abstract	Yes	Limited value	Adds to few MRTs	Narrow	Thin

For peer review only
